# Is there an association between prenatal testosterone and autistic traits in adolescents?

**DOI:** 10.1016/j.psyneuen.2021.105623

**Published:** 2022-02

**Authors:** Niamh Dooley, Amber Ruigrok, Rosemary Holt, Carrie Allison, Alexandros Tsompanidis, Jack Waldman, Bonnie Auyeung, Michael V. Lombardo, Simon Baron-Cohen

**Affiliations:** a Department of Psychiatry, Royal College of Surgeons in Ireland, Dublin, Ireland; b Autism Research Centre, Department of Psychiatry, University of Cambridge, UK; c Department of Psychology, School of Philosophy, Psychology and Language Sciences, University of Edinburgh, UK; d Laboratory for Autism and Neurodevelopmental Disorders, Center for Neuroscience and Cognitive Systems @UniTn,Istituto Italiano di Tecnologia, Rovereto, Italy

**Keywords:** Prenatal testosterone, Amniotic fluid, Fetal development, Puberty, Autism

## Abstract

Prenatal testosterone (pT) is a crucial component in physiological masculinization in humans. In line with the Prenatal Sex Steroid Theory of autism, some studies have found a positive correlation between pT and autistic traits in childhood. However, effects in adolescence have not been explored. Hormonal and environmental changes occurring during puberty may alter the strength or the nature of prenatal effects on autistic traits. The current study examines if pT relates to autistic traits in a non-clinical sample of adolescents and young adults (*N* = 97, 170 observations; age 13–21 years old). It also explores pT interactions with pubertal stage and timing. PT concentrations were measured from amniotic fluid extracted in the 2nd trimester of gestation via amniocentesis conducted for clinical purposes. Autistic traits were measured by self- and parent-reports on the Autism Spectrum Quotient (AQ) which provides a total score and 5 sub-scores (social skills, communication, imagination, attention switching and attention to detail). Self-reported pubertal stage was regressed on age to provide a measure of relative timing. We found no statistical evidence for a direct association between pT and autistic traits in this adolescent sample (males, females or full sample). Exploratory analyses suggested that pT correlated positively with autistic traits in adolescents with earlier puberty-onset, but statistical robustness of this finding was limited. Further exploratory post-hoc tests suggested the pT-by-pubertal timing interaction was stronger in males relative to females, in self-reported compared to parent-reported AQ and specifically for social traits. These findings require replication in larger samples. Findings have implications for understanding the effects of pT on human behavior, specifically existence of effects in adolescence.

## Highlights

• High prenatal testosterone (pT) may be associated with autistic traits in children.
• An association was not observed in our adolescent sample.
• The association between pT and autistic traits may be moderated by pubertal factors.
• Higher pT related to higher autistic traits in adolescents with earlier-onset/faster puberty.
• This interaction of pT and pubertal timing was most evident for social traits.

## 1 Introduction

### 1.1 pT and autistic traits

Autism Spectrum Conditions (henceforth, autism) are the spectrum of phenotypes characterized by differences in social interaction, communication, adjusting to unexpected change, alongside unusually restricted and repetitive behavior, focused or intense interests, and sensory differences. Autism is more commonly diagnosed in males than females (Loomes et al., 2017). In the general population, males also report more autistic traits than females (Baron-Cohen et al., 2001, Greenberg et al., 2018). This has raised the question whether prenatal testosterone (pT), a masculinizing factor, contributes to the development of autism (Baron-Cohen et al., 2011).

Some support for the link between pT and autistic traits has come from several study designs. First, correlations have been reported between pT found in amniotic fluid and parent-reported autistic traits in childhood (Auyeung et al., 2012, Auyeung et al., 2009b, Auyeung et al., 2010), parent-reported empathy, theory of mind (Chapman et al., 2006), restricted interests (Knickmeyer et al., 2005) and early social behaviors (Lutchmaya et al., 2002, Knickmeyer et al., 2005, Knickmeyer et al., 2006b). Amniotic derived pT is particularly valuable as the clinical amniocentesis overlaps with a critical time of gestation when pT levels show sex differences (van de Beek et al., 2004), sexual differentiation of the brain occurs (Goy et al., 1988, Roselli et al., 2011) and autism-related genes show the highest expression (gestational weeks 10–25; Grove et al., 2019). However, one independent study found no relationship between amniotic-derived pT and parent-reported autistic traits (Kung et al., 2016b, Kung et al., 2016a). Differences in sample or methodology may account for the inconsistent findings between Auyeung et al. (2009b) and Kung et al., 2016b, Kung et al., 2016a which both used the Childhood Autism Spectrum Test (CAST) as their outcome.

Second, Congenital Adrenal Hyperplasia (CAH) is an autosomal recessive disorder characterized by cortisol deficiency and elevated androgens beginning prenatally. This elevation is particularly marked in females with CAH. Males with CAH have normal to high androgen concentrations prenatally. Two studies found that girls with CAH score higher on measures of autistic traits than matched relatives (Knickmeyer et al., 2006a, Kung et al., 2016a), however, Kung et al. did not find this difference to be significant and the difference in Knickmeyer et al. were small and driven by one AQ sub-scale (imagination). Engberg et al. (2015) also found that neuropsychiatric diagnoses in general, but not autism specifically, were more common among females with CAH (n = 335; ages 15–37) compared to controls. Finally, Güneş et al. (2020) found that boys (n = 18) but not girls (n = 27) with CAH showed higher autistic traits as measured by the Social Communication Questionnaire compared to non-autistic relatives. The CAH research is therefore mixed regarding increased likelihood of autistic traits.

Third, an association between pT and autism is supported by studies on children of mothers with Polycystic Ovary Syndrome (PCOS), a condition characterized by excess circulating androgens. A meta-analysis found maternal PCOS increases the odds of an autism diagnosis in their children by 66% (Katsigianni et al., 2019), an effect which appears to be independent from genetic factors related to PCOS (Cesta et al., 2020). However children of women with PCOS are also at increased odds of developing other conditions such as ADHD, sleeping, eating and mood-related conditions, which suggests the effect is not specific to autism or more male-skewed conditions (Chen et al., 2020, Berni et al., 2018). Furthermore, no other maternal condition linked with heightened early androgens has been reliably associated with a diagnosis of autism in their children (May et al., 2021).

Fourth, masculinized morphology may be associated with autism. Male-type shifts in the facial morphology of autistic individuals have been reported compared to their non-autistic peers (Tan et al., 2017, Tan et al., 2020a, Tan et al., 2020b) and sex differences in brain structure and function in autistic adults are attenuated, with male-type shifts in specific regions (Floris et al., 2018, Lai et al., 2013). However, not all studies have found associations between male-typical morphology and autistic traits in a direction consistent with increased pT (Kung et al., 2021, Smith et al., 2019, Alaerts et al., 2016, Lee et al., 2020, Bejerot et al., 2012).

The timing of pT measurement may be important. Studies measuring testosterone from umbilical cord blood or infant saliva have not found associations with autistic traits (Whitehouse et al., 2012a, Park et al., 2017, Auyeung et al., 2012, Kung et al., 2016b). Cord-derived pT is also influenced by obstetric noise from labor and glucocorticoid treatment (Keelan et al., 2012). However, the sex difference in umbilical cord testosterone remains significant (Barry et al., 2011) and testosterone levels at this point appear to be relevant to other aspects of child development such as speech and language (Whitehouse et al., 2012b, Hollier et al., 2013).

Two main caveats surround the association between pT and autistic traits. First, studies using the Danish Biobank found no significant difference in amniotic fluid testosterone between autistic and non-autistic boys, showing that other steroid hormones beyond pT are likely involved (Baron-Cohen et al., 2015, Baron-Cohen et al., 2019). This has led to a broadening of the Fetal Testosterone theory of autism to the Prenatal Sex Steroid theory. Second, there has been a shortage of studies linking amniotic-derived pT and autistic traits due to ethical and practical difficulties of obtaining amniotic fluid and as a result most studies have come from one sample pool (Cambridgeshire, UK) and one lab (Xiong and Scott, 2020). Yet pT remains a plausible predictor of autistic traits given (i) the pervasive sex difference in autism (Loomes et al., 2017), (ii) the potential for pT to interact with known likelihood factors for autism, the majority of which also occur in gestation (Emberti Gialloreti et al., 2019) and (iii) the dimensional nature of pT whose distribution across the population may map onto to the autism spectrum. The future of research into pT as a predictor of autistic traits is therefore likely to focus on the interactions between pT and other predisposing factors such as familial/genetic and developmental factors.

### 1.2 pT and autistic traits beyond puberty

One unknown is whether pT exposure can impact behavior beyond childhood and into adolescence. To date, all studies on amniotic pT and autistic traits have assessed outcomes in mostly pre-pubertal children, but it is possible that the hormonal and neuronal changes of puberty could interact with the initial effect of pT on neurodevelopment. Puberty may alter the number or strength of autistic traits, particular social traits. Adolescence is a time of accelerated structural and functional change in the brain, partially mediated by the increase in sex hormones and resulting in a hypersensitivity to social stimuli (Goddings et al., 2012, Blakemore, 2012, Foulkes and Blakemore, 2016). However it is not well understood how puberty affects autistic traits. One longitudinal study of autistic people reported that a sub-group (17%) had significantly poorer outcome after puberty (Billstedt et al., 2005) while another found a general improvement in parent-reported behaviors in their autistic children during adolescence (McGovern and Sigman, 2005).

There is some evidence to suggest the effects of pT on brain structure and function are altered during adolescence. Twins sharing the womb with a male, who are thought to be exposed to higher levels of pT, have larger total brain volumes at age 9 (e.g., females with male twins have larger brain volumes than females with female twins). However, this effect of pT on brain volumes was attenuated and non-significant in adults suggesting it may be developmentally specific (Peper et al., 2009). Beking et al. (2018) found that there was an interaction between amniotic-based pT and pubertal testosterone on a male-typical pattern of neural lateralization during a mental rotation task, which may also indicate a developmentally sensitive effect of pT on brain function. Pubertal timing (in particular early-onset or precocious puberty) has also shown consistent associations with mental health difficulties in adolescence (Dorn, 2007, Kaltiala-Heino et al., 2003, Mendle et al., 2010, Wichstrøm, 2000) and may interact with behavioral effects of pT.

Animal experiments suggest puberty may be a second sensitive period of neuroendocrine organization (Romeo, 2003, Schulz et al., 2009). Manipulation of pubertal testosterone alters sex-typical social and anxiety behaviors in the Syrian hamster (Schulz et al., 2009) and social stressors during puberty (but not in late adolescence) can enhance neurological insults conferred by gestational infection (“two-hit hypothesis”; Giovanoli et al., 2013), a mechanism which some researchers posit may be relevant to autism (Picci and Scherf, 2015).

Taken together, these animal and human studies highlight the importance of taking puberty into account when assessing the behavioral effects of pT in adolescence.

### 1.3 The current study

We test whether pT is related to scores on the Autism Spectrum Quotient (AQ) in individuals aged 13–21. This is a follow-up of the longitudinal study of neurotypical children whose pT was measured from amniotic fluid during gestation and who were assessed for autistic traits as infants (Lutchmaya et al., 2001, Lutchmaya et al., 2002) and children, up to age 10 (Chapman et al., 2006, Knickmeyer et al., 2005, Knickmeyer et al., 2006b, Auyeung et al., 2006, Auyeung et al., 2009b). We assess whether pT is associated with each AQ subscale (social skills, communication, imagination, attention to detail, and attention-switching) as a previous study suggests (Auyeung et al., 2009b). We control for two measures of pubertal development: pubertal stage, whose order is consistent across most individuals, and pubertal timing, which can vary widely between individuals. We hypothesize that pT will be positively correlated with AQ total score, in keeping with previous results in younger children (Auyeung et al., 2009b, Auyeung et al., 2010, Auyeung et al., 2012). We also explore the relative effects of pT on each AQ subscale and whether pubertal factors have any effect on the pT-AQ relationship.

## 2 Material and methods

### 2.1 Participants and procedure

Mothers in Cambridgeshire (UK) who had undergone a routine amniocentesis for clinical purposes (1996–1999) and who gave birth to healthy singletons were invited to take part in a study on the effects of pT on the child’s cognition, behavior and brain development (Baron-Cohen et al., 2004). Those who consented allowed their amniotic fluid to be retrospectively analyzed for testosterone. Children who screened positive for a genetic abnormality at the amniocentesis or later were excluded. Between 2012 and 2018, 105 participants returned for testing, where they completed a range of cognitive tests and questionnaires. Five participants were excluded because of pT values (see below) and a further 3 individuals did not complete an AQ, leaving 97 individuals and 170 AQ score observations for analysis (56 males with 96 AQ scores, 41 females with 74 scores). Demographic details of the sample are provided in Table 1. For participants under 18, parents provided informed consent and participants provided informed assent. Those over 18 provided their own consent and completed only self-report AQ. This study was approved by the Essex 1 National Research Ethics committee.

**Table 1:** Descriptive Statistics for Full Sample and Each Sex.

	Full Sample (N = 97)				Females (n = 41)			Males (n = 56)				Sex Difference (Cohen’s D)^1^	
Variable	**N**	**M**	**SD**	**Min-Max**	**N**	**M**	**SD**		**N**	**M**	**SD**		
pT (nmol/L)	97	0.67	0.47	.10–2.3	41	0.34	0.34		56	0.92	0.41	1.51 * **	
Time of amnio. (weeks’ gestation)	50	16.64	1.48	14–21	15	16.47	1.06		35	16.71	1.64	0.17	
Maternal age	74	35.01	4.74	23–45	31	35.97	4.32		43	34.44	4.96	0.35	
Child age	96	15.61	1.77	13–21	41	15.80	1.78		55	15.46	1.77	0.22	
Pubertal Stage	96	3.30	0.45	1.8–4.0	41	3.61	0.3		55	3.07	0.39	1.55 * **	
Pubertal Timing	96	0.00	0.28	-1.0–0.6	41	0.00	0.26		55	0.00	0.30	< 0.01	
Self-Report AQ													
Total	87	16.32	6.72	4–39	40	14.90	5.67		47	17.53	7.35	0.40	
Social Skill		2.40	2.41	0–10		2.02	2.07			2.72	2.65	0.29	
Communication		2.28	2.03	0–8		1.90	2.01			2.60	2.01	0.35	
Imagination		2.47	1.74	0–7		2.00	1.45			2.87	1.87	0.52 *	
Att. To Detail		5.06	2.16	0–9		5.22	2.11			4.91	2.22	0.14	
Att. Switching		4.11	2.01	0–10		3.75	1.81			4.43	2.13	0.34	
Parent-Report AQ													
Total	86	12.95	8.07	3–40	36	9.11	2.94		50	15.72	9.38		0.89 * **
Social Skill		2.01	2.38	0–10		0.94	0.98			2.78	2.78		0.83 * **
Communication		2.02	2.19	0–10		1.22	1.24			2.60	2.53		0.66 * *
Imagination		2.06	1.96	0–9		0.92	0.94			2.88	2.23		1.15 * **
Att. To Detail		3.99	2.19	0–9		3.89	1.89			4.06	2.39		0.08
Att. Switching		2.87	2.08	0–9		2.14	1.50			3.40	2.29		0.63 * *

*p < 0.05. ** *p* < 0.01. ***p < 0.001.

^1^Significance (*) derived from Welch’s Two-Sample T-Tests (*t*-test variant used for samples with potentially unequal variances).

pT = prenatal testosterone; M = mean; SD = standard deviation

### 2.2 Prenatal testosterone (pT)

**Fig. 1:**
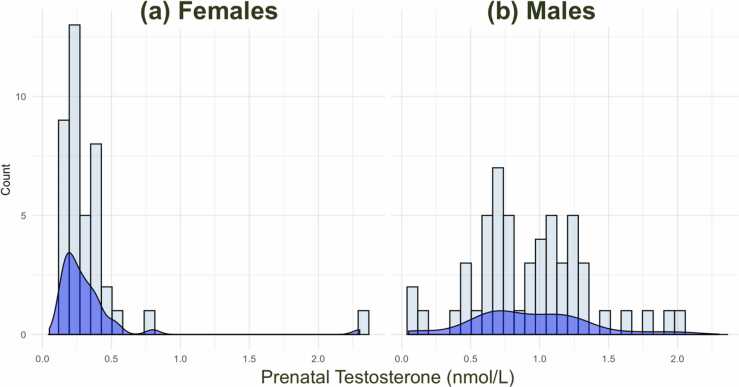
Histogram and density plots for prenatal testosterone (nmol/L) for females (a) and males (b).

PT concentration was measured via radioimmunoassay in amniotic fluid samples collected between 13 and 20 weeks of gestation (Lutchmaya et al., 2001). Amniotic fluid was extracted with diethyl ether, which was evaporated to dryness at room temperature and the extracted material re-dissolved in an assay buffer. Testosterone was assayed by the Count-a-Coat method (Diagnostic Product), which uses an antibody to testosterone coated onto propylene tubes and a 125I-labeled testosterone analog. The detection limit of the assay using the ether-extraction method is 0.05 nmol/L. The coefficient of variation (CV) for between-batch imprecision is 19% at a concentration of 0.8 nmol/L and 9.5% at a concentration of 7.3 nmol/L. The CVs for within-batch imprecision are 15% at a concentration of 0.3 nmol/L and 5.9% at a concentration of 2.5nmol/L. This method measures total extractable testosterone. Five participants were excluded: three participants had undetectable/unreliably low pT levels (<0.10 nmol/L), one sample was unusable, and one was an outlier with a very high pT value (19.50 nmol/L; visible in Fig. 1). As expected, mean pT levels were significantly higher in males compared to females (mean sex difference = 0.57 nmol/L, *t* = −7.66, *p* < 0.001, Cohen’s *d* = 1.51; Table 1) although the distribution of pT values varied considerably between the sexes (Fig. 1). PT was standardized across the full sample.

### 2.3 Autism spectrum quotient (AQ)

Adolescents completed the self-report Adult AQ (Baron-Cohen et al., 2001) while parents completed the Adolescent AQ (Baron-Cohen et al., 2006). Both are 50-item questionnaires with response outcomes on a 4-point scale (definitely disagree, slightly disagree, slightly agree, definitely agree). Item wording was the same across the 2 versions of the AQ, with subject “I” in self-report simply changed to “he/she” in parent-report, allowing direct comparison between them (items listed in Table S10). Approximately half the items are worded to produce a “disagree” response, and half an “agree” response to avoid response bias. Only the valence (and not the strength) of the response was considered when scoring (i.e. slightly and strongly both equal 1 point). Five theoretical sub-scales assess domains of autism-related differences (higher scores corresponding to more differences): social skills, attention to detail, attention switching, communication and imagination (Baron-Cohen et al., 2001). Each domain is assessed by 10 questions giving a maximum total AQ score of 50.

The internal consistency for the AQ total is adequate (range of Cronbach’s alpha across studies 0.67–0.82; Austin, 2005; Hurst et al., 2007). Cronbach’s alpha ranges for sub-scales are as follows: social skill (0.66–0.77), attention switching (0.41–0.67, attention to detail (0.60–0.66), communication (0.47–0.65), imagination (0.40–0.65; Baron-Cohen et al., 2001; Austin, 2005; Hurst et al., 2007).

Participants were required to have either a parent- or self-report AQ (or both) for inclusion. Most (78%) participants had both versions of the AQ, so while the Adult-AQ has only been validated for individuals over 16 (Baron-Cohen et al., 2001) and the Adolescent-AQ has only been validated for those under 16 (Baron-Cohen et al., 2006), most participants had at least one version of the AQ that had been validated for their age-group.

### 2.4 Age

Participant ages ranged from 13 to 21 years (mean = 15.6, SD = 1.77). There was 1 participant with missing age which was imputed with the mean. We refer to all participants as adolescents given recent proposals that the definition of adolescence should be extended to 10–24 years, within which continued biological and social development occurs (Sawyer et al., 2018).

### 2.5 Pubertal stage

The Pubertal Developmental Scale (Petersen et al., 1988) was used to measure pubertal stage. This self-report questionnaire includes a series of items about growth spurt, body hair growth and skin changes for both males and females. Sex-specific questions include facial hair growth and voice change for males, and breast development and the onset of menarche for females. For each item, the participant responds on a 4-point scale: 1 = not started, 2 = barely started, 3 = definitely underway, 4 = seems complete. Onset of menarche was a yes/no question, corresponding to scores of 4/1 respectively. Mean pubertal stage was the average of the 5 items. Adolescent self-reports on this scale have been validated, showing good consistency with parent-ratings on the same scale (Carskadon and Acebo, 1993, Koopman-Verhoeff et al., 2020, Herting et al., 2020), picture-based rating systems (Shirtcliff et al., 2009, Bond et al., 2006, Koopman-Verhoeff et al., 2020) physical examinations by a medical professional (Shirtcliff et al., 2009, Schmitz et al., 2004) and pubertal hormone levels (Shirtcliff et al., 2009, Braams et al., 2015, Herting et al., 2020). This scale also shows excellent internal consistency (Cronbach’s alpha = .93) and test-retest reliability (Intraclass correlation coefficient = 0.87; Koopman-Verhoeff et al., 2020).

### 2.6 Pubertal timing

Pubertal timing was the deviation of an individual’s pubertal stage from the mean stage for their age and sex, within this sample. We regressed pubertal stage on age for males and females separately (standard linear regressions) and the residuals of these tests (Fig. S4) were taken to represent sex-specific variation in pubertal timing. This approach has been used by others to represent pubertal timing (Dorn et al., 2003, Ellis et al., 1999, Ellis and Garber, 2000, Ge et al., 2003, Siegel et al., 1999) and our average trajectories of age and pubertal stage align well with those from other studies (Gunnar et al., 2009, Klump et al., 2007, Smith-Woolley et al., 2017). Negative residuals correspond to developing slower than average; positive residuals correspond to faster development than average. While the terms fast vs slow (pubertal tempo) are used to represent variation in pubertal timing, this variable may also reflect differences in pubertal onset (early vs late onset), which could not be differentiated given cross-sectional measurement of pubertal stage.

### 2.7 Gestational confounds

Certain gestational factors may be associated with pT levels and autistic traits and are therefore potential confounds. The time of amniocentesis (in weeks gestation) for instance is correlated with autistic traits in infants (Auyeung et al., 2010, Knickmeyer et al., 2005). Unfortunately, due to this data not being systematically recorded at the first wave of data collection, it was available for only a sub-sample (n = 50). Maternal age at birth (in years) was available for more participants (n = 74) and was also controlled for due to previously reported associations with pT levels (Auyeung et al., 2012) and sex-typical behavior in children (Auyeung et al., 2009a).

### 2.8 Statistics

#### 2.8.1 General approach

All analyses were performed in R (R Core Team, 2012). Linear mixed models were used to predict AQ scores using the *lmer* function from the *lme4* package (Bates et al., 2014). Parameters were estimated using REML (Restricted Maximum Likelihood). Both parent- and self-reported AQ scores were considered dual observations of the same latent construct (AQ score), which was achieved by including a random intercept for each subject (1|ID) and including rater a within-subject variable. Binary variables were centered (sex, rater; contrasts of −0.5 and 0.5). PT was standardized (centered and scaled) while pubertal stage was centered to retain original scale.

While linear mixed models are robust to violations of distributional assumptions (Schielzeth et al., 2020), we also ran generalized linear mixed models using a gamma distribution to account for the positive skew in AQ scores (details in Supplementary Material).

A power analysis, using R package *simr*, determined that our linear mixed model and sample size (*n* = 97, number of observations =170) had sufficient power (83%) to detect a main effect of pT on AQ similar in magnitude to that found in younger children (Auyeung et al., 2009b). Large (*β* = 12), moderate (*β* = 6) and small (*β* = 3) interactions between pT and our pubertal measures could be observed with power of 100%, 60% and 20% respectively. The interaction between pT and sex was likely to be underpowered (required sample size for 80% power estimated between 320 and 2000 participants). Allowing estimates to vary by sex is crucial to any test of testosterone effects, therefore we could not exclude the pT x sex interaction despite power issues. Details of power calculations are described in Supplementary Material.

We calculated 95% confidence intervals for the Pearson’s correlations between pT and AQ scores from our data and that of (Auyeung et al., 2009b) using the Fisher’s r-to-z transformation.

#### 2.8.2 Predicting AQ total

Predictors included sex (m/f), rater (parent/self), (and continuous variables) pT, pubertal stage, pubertal timing, maternal age at birth and time of amniocentesis. To avoid multicollinearity between pubertal stage and pubertal timing (due to the latter being generated from the former), two separate models were run. The pubertal stage model included 4 main effects (pT, sex, rater, pubertal stage) and 2 interactions (pT-by-sex, pT-by-pubertal stage) at baseline. The pubertal timing model simply replaced pubertal stage with pubertal timing. Maternal age and time of amniocentesis were added in consecutive models (M2, M3) as their inclusion reduced the sample size to 74 and 50 respectively. Inclusion of a pT x sex interaction allows the slope of the pT-outcome relationship to differ for males and females which was deemed necessary given differences in pT distribution observed across sex (Fig. 1) and the potential for sex differences in androgen signaling. Inclusion of interactions between pT and puberty measures allowed us to explore whether the effects of pT on AQ changed across pubertal maturation (pubertal stage) or whether the effect differed between on average “fast” and “slow” developers (pubertal timing). Our hypothesis was supported if we found a significant effect of pT on AQ, or an interaction of pT-by-sex on AQ (such that the correlation existed in one sex but not the other). If no association was observed, equivalence tests were carried out to support absence of a meaningful effect with regards to an a priori effect size (Lakens, 2017). Given that there were 3 pT-related effects of interest (main effect of pT, interaction of pT-by-sex and interaction of pT-by-pubertal stage/timing), we applied a more conservative Bonferroni-adjusted threshold of significance (0.05/3 = 0.0167) when interpreting results of this model.

We tested whether the relationship between pT and AQ total score was better described as quadratic (versus linear). An pT^2^ term was included as an additional fixed effect. A quadratic effect of pT on AQ total would be supported if the model including a quadratic term was a better model fit to the data than the model including linear terms only (according to Log Likelihood, AIC & BIC) and if the pT^2^ term itself was significant.

Sensitivity analyses were performed to verify results of the linear mixed models: (1) simpler models predicting AQ from pT (less covariates and rater-stratified models) (2) sex-stratified analyses (3) generalized linear mixed models to account for violations of assumptions of normality and homoscedasticity and (4) a repeat of main analyses without participants with an autism diagnosis (n = 1).

#### 2.8.3 Predicting AQ sub-scores

The five AQ subscales were dependent variables in five separate tests. We had no specific hypotheses about the how pT would relate to the AQ subscales therefore, on the basis of AQ total results, we included pubertal timing only (not pubertal stage) in prediction models of AQ subscales to restrict the number of tests performed. The baseline model (M1) used to predict each AQ subscale therefore included 4 main effects (pT, sex, rater, pubertal timing) and 2 interactions (pT-by-sex, pT-by-pubertal timing). In subsequent models, we also controlled for maternal age at birth (M2) and time of amniocentesis (M3). We report Bonferroni corrected significance levels (*p* = .01) for reference (0.05/5 sub-scales). We also conducted sensitivity analyses with these sub-score results (as in 2.8.2).

## 3 Results

### 3.1 Descriptive Statistics

Table 1 shows that there were significant sex differences in pT levels (males > females), pubertal stage (females > males), parent-reported AQ totals (males > females) and the self-reported AQ subscale of imagination (males > females). Whilst self- and parent-ratings on the AQ total were significantly correlated (*r*(74) = .42, *p* < .001), there was also a significant difference whereby adolescents rated themselves higher than parents on average (*t*(75) = −4.38, *p* < .001; Table 1). Maternal age at birth was significantly correlated with pT (*r* (71)= −0.35, *p* = .002) but not AQ totals. Time of amniocentesis was weakly correlated with both amniotic pT levels (*r* (48) = 0.24, *p* = 0.10) and rater-averaged AQ totals (*r* (48)= −0.27, *p* = .05).

### 3.2 Inferential statistics

#### 3.2.1 pT and AQ Total

Using linear mixed models, no significant main effect of pT on AQ total was observed when controlling for sex, pubertal stage/timing, AQ rater (M1), time of amniocentesis (M2) and maternal age (M3). No significant interaction between pT and sex was observed either (Table 2). The Pearson’s correlation between pT and rater-averaged AQ totals for males was *r* = −0.11 (*p* = .44, *n* = 56) and for females was *r* = 0.17 (*p* = .30, *n* = 41). No significant correlation was observed between pT and specifically parent-reported AQ total (male *r* = −0.07, *p* = 0.65; female *r* = 0.08, *p* = 0.65) or self-reported AQ total (male *r* = −0.15, *p* = 0.32; female *r* = 0.23, *p* = 0.15). The quadratic relationship between pT and AQ total was not significant i.e., the pT^2^ term was not significant and its inclusion did not improve model fit (Table S1). The hypothesis that pT would correlate positively with AQ total was therefore not supported. The 95% confidence interval for the correlation between pT and rater-averaged AQ scores (CI = −0.03 to 0.36, *r* = 0.17, *p* = 0.09) only overlapped to a small extent with the interval previously reported in younger children (CI = 0.30–0.51, *r* = 0.41, *p* < 0.01; Auyeung et al., 2009b). Sensitivity analyses, specifically a set of simpler models (Tables S3-5), sex-stratified analyses (Table S6-7) and a generalized linear mixed model (Table S8), supported this null finding. Using Auyeung et al. (2009)’s point estimate (r = .41) as the upper bound of an equivalence test, we found that our observed effect size (on both parent-reported AQ and rater-averaged AQ scores) were significantly within the equivalent bounds. Our observed effect was therefore not equal to, or greater than, the point estimate reported by Auyeung et al. (2009).

**Table 2:** Predicting AQ total. Fixed effect estimates *β* (and standard errors). P-values derived from Wald tests. M1-M3 denote models adjusting for increasing numbers of covariates. Pubertal stage and timing models split to avoid multicollinearity.

		Models with pubertal stage			Models with pubertal timing		
		M1 Baseline model	M2 M1 + maternal age	M3 M2 + time of amniocentesis	M1 Baseline model	M2 M1 + maternal age	M3 M2 + time of amniocentesis
M1	pT	-0.97(1.76)	-0.33(1.95)	-1.87(4.31)	-1.04(1.74)	-0.43(1.92)	-1.98(4.20)
		*p = 0.59*	*p = 0.87*	*p = 0.67*	*p = 0.56*	*p = 0.83*	*p = 0.64*
	Sex	7.48(3.19)	5.22(3.46)	5.64(8.47)	6.11(3.01)	4.33(3.24)	6.38(7.84)
		*p = 0.02*	*p = 0.14*	*p = 0.51*	*p = 0.05*	*p = 0.19*	*p = 0.42*
	Rater	-3.61(0.83)	-3.53(0.91)	-3.09(1.25)	-3.67(0.83)	-3.54(0.92)	-3.13(1.26)
		*p < 0.001*	*p < 0.001*	*p = .02*	*p < 0.001*	*p < 0.001*	*p = 0.02*
	pT *x* Sex	1.72(3.67)	2.15(3.94)	9.50(8.48)	-0.60(3.49)	-1.00(3.71)	3.47(8.43)
		*p = 0.64*	*p = 0.59*	*p = 0.27*	*p = 0.87*	*p = 0.79*	*p = 0.69*
	Pubertal Stage	2.59(1.79)	1.56(2.00)	-1.67(2.62)	–	–	–
		*p = 0.15*	*p = 0.44*	*p = 0.53*			
	pT *x* Pubertal Stage	3.94(2.42)	5.65(2.61)	10.19(3.31)	–	–	–
		*p = 0.11*	*p = 0.04*	*p = 0.003*			
	Pubertal Timing	–	–	–	2.28(2.14)	2.06(2.21)	-1.05(2.63)
					*p = 0.29*	*p = 0.36*	*p = 0.69*
	pT *x* Pubertal Timing	–	–	–	5.98(2.58)	7.28(2.74)	11.15(3.27)
					*p = 0.02*	*p = 0.008*	*p = 0.001*
M2	Maternal Age at Birth	–	-0.01(0.16)	-0.12(0.20)	–	0.03(0.16)	-0.05(0.20)
			*p = 0.96*	*p = 0.56*		*p = 0.83*	*p = 0.79*
M3	Time of Amniocentesis (gestational weeks)	–	–	-2.07(0.61)	–	–	-2.02(0.59)
				*p = 0.001*			*p = 0.001*
	# Observations	170	139	94	170	139	94
	# Participants	95	73	49	95	73	49
	Log Likelihood	-551.02	-446.96	-297.37	-549.91	-445.71	-296.48
	AIC	1120.04	913.91	616.74	1117.83	911.42	614.95
	BIC	1148.26	943.26	644.72	1146.05	940.77	642.93

pT = Prenatal testosterone; AIC = Akaike Information Criterion; BIC = Bayesian Information Criterion.

Bonferroni-adjusted *p*-threshold = .0167

**Fig. 2:**
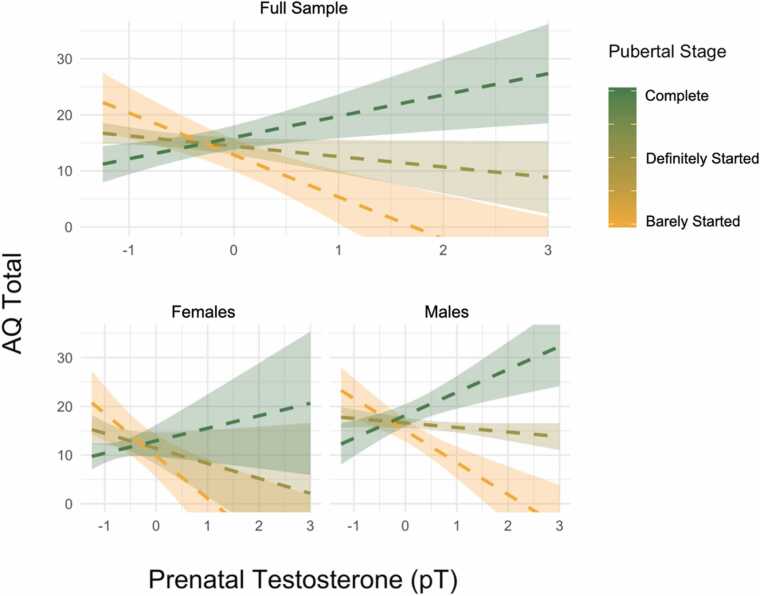
Effects of pT on AQ totals for 3 different stages of pubertal development. Full sample results (top) and sex-stratified (bottom).

Note: Three discrete scores along the continuous Pubertal Development Scale reflect puberty that has “barely started” (2), “definitely started” (3), and is “complete” (4). AQ values on the y-axis capture the average of parent- and self- reported AQ. Note higher scores on the AQ correspond to more autistic traits. Error bands refer to standard errors. Estimates taken from M2

Table 2 shows the interaction between pT and pubertal *stage* on AQ totals was not significant at the Bonferroni-corrected threshold (0.0167) in the baseline model M1 or after adjusting for maternal age (M2) but was significant in M3, adjusted for both maternal age and time of amniocentesis (*β* = 10.19, *SE* = 3.31, *t* = 3.08, *p* = .003). Fig. 2 suggested that the correlation between pT and AQ total became increasingly positive the later the pubertal stage. Sensitivity analyses suggested this interaction was stronger in males (Tables S6-7) and in self-reported AQ scores (Tables S4-5) but generalized linear mixed models found the interaction was not significant in any model (M1-M3), limiting the robustness of this finding.

**Fig. 3:**
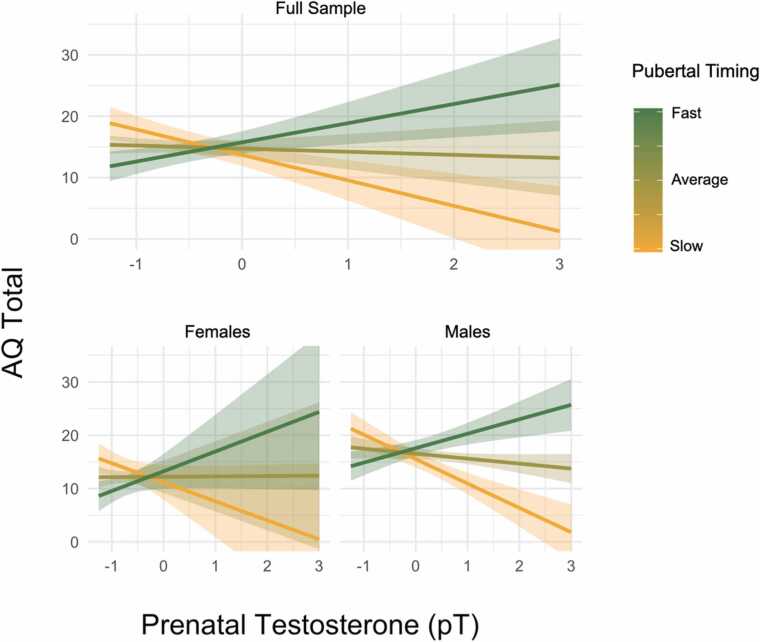
Effects of pT on AQ totals for 3 levels of pubertal timing. Full sample results (top) and sex-stratified (bottom).

Note: Lines represent three levels of pubertal timing: 0.5 (“fast”), 0 (“average”) and -0.5 (“slow”). AQ values on the y-axis capture the average of parent- and self- reported AQ. Note higher scores on the AQ correspond to more autistic traits. Error bands refer to standard errors. Estimates from M2.

Table 2 also shows an interaction between pT and pubertal timing on AQ total which was significant at the Bonferroni-corrected threshold after correction for maternal age (M2: *β* = 7.28, *SE* = 2.74, *t* = 2.65, *p* = 0.01; *N* = 73) and time of amniocentesis (M3: β = 11.15, *SE* = 3.27, *t* = 3.41, *p* = 0.001; *N* = 49) but not in the baseline model M1. Fig. 3 shows that pT was positively associated with AQ total for individuals who were fast developers (i.e. advanced pubertal stage for their age relative to the rest of this sample). Those who were ‘slow developers’ (i.e. relatively under-developed for their age) showed a negative correlation between pT and AQ total. Those developing at an average speed for their age showed a weak negative correlation between pT and AQ. This interaction was also subjected to several sensitivity analyses: rater-split tests showed the interaction was stronger for self-reported AQ than parent reports (Table S4-5); sex-stratified analyses showed the interaction was strongest in males (Table S6-7; Fig. S2) and generalized linear mixed models showed the interaction to be attenuated and non-significant at the corrected threshold once the skew in outcome was accounted for (Table S8). On balance, the reliability of this interaction should thus be interpreted with caution given the lack of guiding hypothesis, low power, and due to lack of support from alternative modeling (generalized linear mixed analysis). However, for explorative purposes we investigated the pT *x* pubertal timing interaction among the AQ subscales.

Of all predictors of AQ total, rater had the most reliable effect. On average, adolescents rated themselves over 3.0–3.7 points higher than their parents did on the 50-point AQ scale (Table 2). Sex also had a significant effect in M1 with males having higher AQ totals than females, but this became non-significant after controlling for maternal age at birth (M2).

#### 3.2.2 pT and AQ subscales

**Fig. 4:**
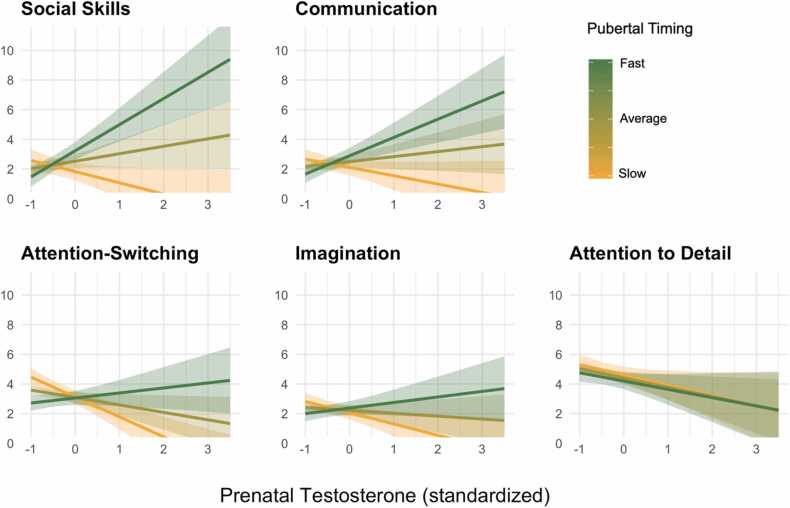
Effects of pT on the AQ subscales for 3 levels of pubertal timing.

Note: Lines represent three pubertal timing subgroups: 0.5 (“fast”), 0 (“average”) and − 0.5 (“slow”). AQ values on the y-axis capture the average of parent- and self- reported AQ. Error bands refer to standard errors. Estimates from M2 models tested in the full sample.

To limit the number of tests performed on AQ subscales, we explored the pubertal timing model only, as pubertal timing had shown more reliable interaction effects with pT on AQ total score, and as pubertal variables were highly correlated. Fig. 4 depicts the interaction between pT and pubertal timing on each AQ subscale. The interaction between pT and pubertal timing was strongest for social skill, and this effect was significant in the baseline model (M1) and after controlling for gestational confounds (M2, M3) at a corrected p-threshold (M1: *β* = 2.20, *SE* = 0.83, *p* = 0.008; M2: *β* = 2.52, *SE* = 0.90, *p* = 0.006; M3: *β* = 4.24, *SE* = 1.14, *p* < 0.001). Fig. 4 shows, as with the AQ total, that pT was positively associated with social skill differences in individuals who went through puberty faster than average, and pT was inversely associated with these differences in those who progressed through puberty slower than average. For those with average pubertal timing for their sex and age, there was little correlation between pT and social skills. Fig. S1, suggested this interaction was more reliable in males compared to females, an observation which was supported by sex-stratified results (Tables S6-7). Generalized mixed models found the interaction between pT and pubertal timing on social skills to be significant at corrected thresholds across all levels of covariate adjustment (M1-M3; Table S9), supporting the robustness of this finding to model variation. Full AQ subscale results are provided in Table S2.

An interactive effect of pT and pubertal timing was also observed for the communication subscale, though it was only significant at the corrected threshold for the fully-adjusted model in the restricted sample (M3; n = 49) in both linear mixed models (Table S2; M3: *β* = 3.34, *SE* = 0.86, *p* < 0.001) and generalized linear mixed models (Table S9; M3: *β* = 2.09, *SE* = 0.72, *p* = 0.006), therefore this result should be interpreted with caution.

No significant main effects of pT were observed for any subscale.

There was just one individual in the analysis who had been diagnosed with an autism spectrum condition. All results remained highly similar when this individual was excluded (Supplementary Material, Sensitivity Analysis 4).

## 4 Discussion

This is the first study to examine the effect of amniotic-derived pT levels on autistic traits in adolescence and the first to test interactions between pT and pubertal development. We did not find a positive correlation between pT and autistic traits (measured by the AQ) in the full sample, or either sex. A number of sensitivity analyses verified this null finding (Table S3-S8; Figs. S2-S3) and equivalence tests supported the absence of a meaningful effect. Exploratory findings indicated that the effects of pT were moderated by pubertal timing, particularly in males, such that having both high pT and early/fast pubertal development was linked with the highest AQ scores while having both high pT and slow/late puberty was linked with lowest AQ scores (Fig. 3). However, the evidence supporting this interaction was preliminary, had limited statistical robustness and requires replication. Further exploratory analysis of the AQ subscales suggested that this interaction between pT and pubertal timing was most relevant to social skills (Fig. 3).

### 4.1 Direct effect of pT on autistic traits

The lack of association between amniotic pT and AQ contrasts with some previously reported positive correlations between pT and autism-related traits in infants (Lutchmaya et al., 2001, Lutchmaya et al., 2002, Auyeung et al., 2010) and young children (Chapman et al., 2006, Auyeung et al., 2006, Auyeung et al., 2009b, Auyeung et al., 2012, Knickmeyer et al., 2005, Knickmeyer et al., 2006b). Most relevantly, a positive correlation between pT and parent-report AQ (total score and sub-scores) was observed in children aged 6–10 (Auyeung et al., 2009b), some of whom also took part in the current study. Given the Auyeung et al. study and ours were based on largely overlapping samples and both used the AQ to measure autistic traits, differences in results could have arisen from differences in sample size (*n* = 97 Vs Auyeung et al., 2009b *n* = 235) or sample age (~ 16 years Vs ~ 9 years in Auyeung et al., 2009b). A power analysis determined our study had sufficient power (83%) to detect an effect similar in magnitude to that found in Auyeung et al., 2009b (see Methods 2.7.1) and our results do not indicate even a weak positive association (Table 2) therefore differences in results between our study and those of Auyeung et al. (2009b) are more likely to have arisen from differences in the age/developmental stage of the participants.

It may be that increased peer comparison and social motivation during adolescence alters how individuals score on the AQ. There have been no longitudinal studies of AQ across adolescence however puberty has been shown to parallel significant changes in social emotion processing which may counteract, or at least interact with, autistic traits (Goddings et al., 2012).

The prenatal sex steroid theory of autism does not make explicit hypotheses about the nature of effects into, or beyond puberty, as this has never been tested, however our results suggest the positive correlation between pT and autistic traits found in pre-pubertal children may not generalize to adolescents. Current theories may need to be revised to acknowledge possible developmentally dependent effects of prenatal hormones on autism-related traits. Future studies should investigate whether the association between pT and autistic traits remains null into adulthood and how associations between other steroids (e.g. estrogens) and autistic traits (Baron-Cohen et al., 2019, Tsompanidis et al., 2021) are affected by adolescence.

### 4.2 Interaction of pT and puberty on autistic traits

Fig. 3 shows the interaction between pT and pubertal timing, in which pT levels are positively correlated with AQ total scores for those who experienced puberty relatively faster than their peers, while pT is inversely correlated with AQ total scores in those with slow (or delayed) puberty. The interaction was stronger in self-reported AQ than parent-reported AQ (Tables S4-5) and was driven by males (Table S6-7; Fig. S2). Analysis of the AQ sub-scales suggest the interaction may apply to social skills (Fig. 4) more than the sum of all traits (AQ total). However, the robustness of interactions between pT and pubertal variables (stage and timing) were not supported by generalized linear mixed models which accounted for the non-normally distributed outcome (AQ) and residuals. Our analysis was also likely underpowered (~60%, supplementary material) to detect such interaction effects.

Findings from other human studies support the plausibility of our trend interaction between pT and pubertal timing on autistic traits. One study in 14–16-year-old adolescents showed prenatal and pubertal testosterone levels had an interactive effect on male-typical patterns of brain lateralization during a visuospatial task (Beking et al., 2018). Specifically, in those with low pT exposure, pubertal testosterone increased or “masculinized” lateralization while it decreased or “de-masculinized” lateralization in those with high pT. While Beking and colleagues measured pubertal testosterone rather than pubertal timing (although they are likely correlated; Marceau et al., 2019; Shirtcliff et al., 2009), both our findings and those of Beking et al. support the idea that the effects of pT may interact with pubertal factors to influence sexually differentiated traits. Peper et al. (2009) found that putative effects of pT due to having a male twin in utero were seen in brain volumes in children aged 9 but not in adults, suggesting that the effect of pT on brain volume does not persist beyond adolescence.

It may be that both high pT and early/fast pubertal timing are by-products of a polygenetic tendency to autism. The majority of genetic contribution to autism is likely due to common variants (Gaugler et al., 2014) which may influence both the production of fetal steroids and pubertal timing. Alternatively, pubertal timing may be an indirect measure of the prenatal endocrine environment and the interaction between pT and pubertal timing may capture interaction of multiple steroids or chronicity of high pT throughout gestation. Of potential relevance, pubertal factors only had a statistical effect on AQ scores for higher levels of pT levels (Fig. 2, Fig. 3, Fig. 4). In animal models, prenatal administration of testosterone and glucocorticoids delay the onset of puberty (Cruz and Pereira, 2012, Smith and Waddell, 2000, Abbott et al., 2009, Padmanabhan et al., 2006) and prenatal blocking of androgen signaling (via flutamide) results in earlier onset of puberty in rhesus monkeys (Herman et al., 2006). In humans, females with PCOS are more likely to have been exposed to high pT in utero (Risal et al., 2019) and to experience earlier onset of puberty (Ibáñez et al., 2009). Prenatal exposure to endocrine-disrupting chemicals from common household and hygiene products (e.g. pthalates; Harley et al., 2018) can also alter pubertal timing.

Table 2 shows that the *main* effect of pubertal timing on AQ total was not significant. Our results may therefore be consistent with a “double-hit” model of autistic traits (Picci and Scherf, 2015) in which the combination of a gestational hit (high pT levels) and an adolescent hit (pubertal development earlier/faster than average) is linked with the highest AQ scores.

### 4.3 Social Vs non-social subscales

Amongst the 5 sub-domains of the AQ, the largest pT-by-pubertal timing interaction was observed for social skills (Fig. 4). The social skills scale of the AQ consists of items such as “*I prefer to do things with others rather than on my own*” and “*I find it hard to work out people’s intentions*” (Table S10). This effect was significant at the Bonferroni-corrected alpha level in all levels of adjustment of the linear mixed model (M1-M3; Table S2) and in sensitivity analyses which accounted for the skew in AQ scores (generalized linear mixed models M1-M3; Table S9). As with the AQ total, the interaction was stronger in males compared to females (Fig. S1; Tables S6-7). Weaker interactive effects were observed for communication and attention-switching which were not significant at the corrected threshold. Social and non-social aspects of autism have been shown to be dissociable on the basis of genetics (Warrier et al., 2019, Happé and Ronald, 2008) and factor analysis (Hoekstra et al., 2008). The stronger interaction effects of pT on social aspects of autism is consistent with a neuroimaging study on a sub-group of our sample (Lombardo et al., 2018). It suggested a stronger effect of pT on social brain networks (default mode network) in adolescence than other networks underlying emotion, reward,or language. While the interaction effect of pT and pubertal timing on social skills needs to be replicated in larger cohorts, our results provide preliminary evidence that pT and pubertal factors may be relevant to social aspects of autism, particularly in males.

### 4.4 Rater discordance

AQ scores varied significantly between parent- and self-reports, which is often the case for reports of mental health in older adolescents (Stratis and Lecavalier, 2015). Despite equivalency of items and scoring methods across versions, adolescents rated themselves 3–4 points higher on the AQ (range 0–50) than their parents (Table 2) and this rater-based discrepancy was stronger for females (e.g., rater *β* = 5.73–5.91) than males (rater *β* = 1.67–1.96; Table S6-7). This sex difference may be explained by under-reporting of female autistic traits by parents due to “camouflaging” (e.g. Kenyon, 2014). The extent of rater discordance on the AQ highlights the importance of including multiple reports for this age-group. To improve construct validity even further, we recommend that future studies incorporate expert-rated assessments of autism spectrum traits.

### 4.5 Strengths and limitations

Limitations include the non-exhaustive list of autism-linked traits included in the AQ and the relatively small sample size limited our range of pT values. In particular, female pT values were low with narrow distribution (Fig. 1), making it difficult to conclude whether there was no female effect or whether the pT variance was too small to detect effects. A second limitation is our singular estimate of T from amniotic fluid, which represents only one steroid analyte within a defined time window. Rodeck et al. (1985) also found that amniotic pT did not correlate with fetal serum pT taken around the same time. Given reported effects of other steroid hormones (e.g. estrogens) on childhood autism (Baron-Cohen et al., 2015, Baron-Cohen et al., 2019, Tsompanidis et al., 2021), future studies using amniotic-derived pT should aim to measure a range of steroid hormones. Third, ascertainment bias may be present and limit the generalizability of these results, as the women referred to amniocentesis between 1996 and 1999 are unlikely to represent the typical population of expectant women. Fourth, our sample overlaps with those previously studied (Lutchmaya et al., 2001, Lutchmaya et al., 2002, Auyeung et al., 2006, Auyeung et al., 2009b, Chapman et al., 2006, Knickmeyer et al., 2005, Knickmeyer et al., 2006b), although this is the first study of the pT-autistic traits link in the cohort during adolescence (Lombardo et al., 2018). The shortage of data from other cohorts is a limitation to the generalizability of these findings. Finally, pubertal development was measured by self-report only and while this measure has shown good consistency with more objective measures and parent-ratings (see Section 2.5) some measurement error likely remains.

A core strength of this study is its use of amniotic-derived pT, a direct measure of a specific hormone at a defined window of gestation rather than a retrospective proxy. Amniotic data is an increasingly valuable resource as the amniocentesis procedure has been slowly phased out in favor of serum karyotyping and ultrasound examinations. Other strengths of the study are the inclusion of dual reports of autistic traits (doubling observations and allowing inclusion of individuals with either observation) and the inclusion of multiple trait scales (communication, attention-to-detail, etc.). Finally, the use of multiple statistical models with different underlying assumptions (linear mixed models; generalized linear mixed models; sex-stratified tests) allowed us to probe the robustness of findings.

### 4.6 Conclusion

This is the first study to investigate behavioral correlates of mid-gestational pT in adolescents and young adults, and the first to assess the contribution of puberty. Our findings suggest that the positive correlation between pT and autistic traits, previously observed in infants and children of the same cohort, does not persist into adolescence. However, an exploratory analysis suggested pT and pubertal timing may have interactive effects on autistic traits. This interaction was stronger for social aspects of autism and for males. This finding was explorative and additional research in both humans and animals is needed to test its robustness. Our results also highlight the importance of including pubertal measures and multi-rater behavioral assessments when investigating hormone-behavior links in adolescents.

## Author Note

The data used in this report were from a longitudinal study led by the Autism Research Center in Cambridge. The authors have no conflicts of interest to disclose.
