# Supplementary material for: Is there an association between prenatal testosterone and autistic traits in adolescents?
Source: Psychoneuroendocrinology. 2022 Feb;136:105623. doi: 10.1016/j.psyneuen.2021.105623 (PMC8783053; doi:10.1016/j.psyneuen.2021.105623)
Supplement: Supplementary file 1 — Supplementary material [file mmc1.docx]

Supplementary Material

**Power Analysis**

Auyeung et al. (2009b) had shown that 1 unit increase of pT (nmol/L) led to a 11.61 - 11.92 increase in AQ total (controlling for sex and the presence of an older sibling) in a sample of 6-10-year-old children which partially overlapped with our own. We had standardized pT so that its unit was the sample standard deviation (where 1 SD = 0.47 nmol/L; [Table 1](#Table1)), the effect size reported in Auyeung et al. (2009) therefore needed to be roughly halved for an estimate of how 1 SD of pT would relate to AQ total score. A conservative effect size of a 5 was therefore used in the power analysis (i.e. 5-point increase in AQ for each 1 SD increase in pT). Simulation-based power analysis was performed using the powerSim function from the *simr* package (test = z-test, number of simulations = 500, alpha= 0.05, number of subjects = 97, number of observations = 170, seed = 123). Power to detect a main effect of pT (with effect size *B* = 5) in M1 was shown to be 81% (95% CI = 78-85%). In M2, after inclusion of maternal age and drop in number of observations to 94, power to detect this main effect decreased to 75% (95% CI = 71- 79%) and M3’s power to detect this main effect decreased to 23% (19-26%). However, as time of amniocentesis was a potentially important confound to include we included M3 in results but urged caution against interpreting M3 results in isolation.

Power analyses were also performed to detect power for interactions at various projected effect sizes (e.g. small, medium and large) with the given sample.

For the interaction between pT and pubertal timing/stage (measured on the z-scale), one scenario was that pT might correlate positively with AQ scores only for those with the highest values on these variables, that for each unit (SD) change (e.g. from -0.5 to 0.5 of pubertal timing), the slope between pT and AQ increases by magnitude of X. Using the *rsim* package, we calculated that large (X=12), moderate (X=6) and small (X=3) changes to the slope would necessitate samples sizes of roughly 40, 130 and 600 to achieve 80% power. With our sample size (n=97), we would have 99% [95% CI = 98-100%], 65% [61-70%] and 22% [19-26%] power to detect the interaction at these large, moderate and small effect sizes respectively in the baseline model M1.

Regarding the pT by sex interaction, one scenario was that males would fully account for the association between pT and AQ (“knockout” interaction), the interaction term might have a similar value as the predicted main effect (i.e. a 1 SD change in male pT results in 5-point change in AQ) but 1 SD change in female pT results in 0-pt change in AQ. In this scenario, we would have ~25% power to observe the interaction effect with our sample size (n=97; # observations=170) in the baseline model M1. To achieve 80% for that scenario, the required sample size was over 320 participants. An alternative scenario would be that the pT-AQ association was positive for both sexes but stronger in one sex (e.g. 50% attenuation) , we might observe an interaction beta of 2 (e.g. males have a slope twice the magnitude of females). We would have 10% power to detect this interaction with our sample size. To achieve 80%, we would require over 2,000 participants.

Note that estimate values (e.g. “X=6”) are in terms of the unstandardized beta from the mixed model and refer to original units of the AQ.

Additional Results

**Table S1.** Linear Vs. quadratic effects of pT on AQ total. No pT^2^ term was significant and model fit statistics show no improvement as a result of including pT^2^ (in comparison to models containing linear pT term only; Table 2). Other covariates included in models (coefficients not reported) included; sex, rater and pubertal stage/timing (M1), maternal age at birth (M2) and time of amniocentesis (M3).

|  | Model with pubertal stage | | | |  | Model with pubertal timing | | | | |
| --- | --- | --- | --- | --- | --- | --- | --- | --- | --- | --- |
|  | M1  Baseline model | M2  M1 + maternal age | M3  M2 + time of amniocentesis | |  | M1  Baseline model | | M2  M1 + maternal age | M3  M2 + time of amniocentesis | |
| pT | -0.89(1.78) | -0.07(1.97) | -2.12(4.32) | |  | -1.00(1.76) | | -0.26 (1.94) | -2.08(4.26) | |
|  | *p = 0.62* | *p = 0.98* | *p = 0.63* | |  | *p = 0.58* | | *p = 0.90* | *p = 0.63* | |
| pT^2^ | -0.37(0.78) | -0.78(0.79) | -0.87(0.99) | |  | -0.19(0.76) | | -0.51(0.78) | -0.19(1.01) | |
|  | *p = 0.63* | *p = 0.33* | *p = 0.38* | |  | *p = 0.81* | | *p = 0.52* | *p = 0.85* | |
| # Observations | 170 | 139 | 94 | |  | 170 | | 139 | 94 | |
| # Participants | 95 | 73 | 49 | |  | 95 | | 73 | 49 | |
| Log Likelihood | -550.24 | -445.79 | -296.07 | |  | -549.24 | | -444.82 | -295.53 | |
| AIC | 1120.48 | 913.58 | 616.14 | |  | 1118.47 | | 911.65 | 615.06 | |
| BIC | 1151.84 | 945.86 | 646.66 | |  | 1149.83 | | 943.93 | 645.58 | |
| AIC = Akaike Information Criterion; BIC = Bayesian Information Criterion; pT = Prenatal testosterone | | | |  |  |  |  | | |  |

**Table S2.** Fixed effect estimates *β* (and standard error) from predictors of AQ subscales. P-values derived from Wald tests.

|  | Social Skills | | | Communication | | |
| --- | --- | --- | --- | --- | --- | --- |
|  | M1  Baseline model | M2  M1 + maternal age | M3  M2 + time of amniocentesis | M1  Baseline model | M2  M1 + maternal age | M3  M2 + time of amniocentesis |
| **pT** | 0.32(0.56) | 0.59(0.63) | 0.06(1.43) | 0.09(0.48) | 0.41(0.55) | 0.43(1.14) |
|  | *p = 0.57* | *p = 0.35* | *p = 0.97* | *p = 0.86* | *p = 0.46* | *p = 0.71* |
| **Sex** | 0.82(0.96) | 0.22(1.06) | 0.81(2.66) | 0.87(0.82) | 0.28(0.94) | 0.14(2.13) |
|  | *p = 0.40* | *p = 0.84* | *p = 0.77* | *p = 0.30* | *p = 0.77* | *p = 0.95* |
| **pT *x* Sex** | -0.82(1.12) | -1.12(1.22) | -0.03(2.87) | -0.90(0.95) | -1.02(1.07) | -0.39(2.29) |
|  | *p = 0.47* | *p = 0.36* | *p = 1.00* | *p = 0.35* | *p = 0.34* | *p = 0.87* |
| **Rater** | -0.48(0.26) | -0.49(0.29) | -0.28(0.38) | -0.37(0.26) | -0.25(0.30) | -0.42(0.39) |
|  | *p = 0.08* | *p = 0.09* | *p = 0.47* | *p = 0.17* | *p = 0.41* | *p = 0.30* |
| **Pubertal Timing** | 1.27(0.68) | 1.41(0.72) | -0.14(0.92) | 0.81(0.58) | 0.78(0.64) | 0.10(0.69) |
|  | *p = 0.07* | *p = 0.06* | *p = 0.88* | *p = 0.17* | *p = 0.22* | *p = 0.89* |
| **pT *x* Pubertal Timing** | 2.20(0.83)** | 2.52(0.90)** | 4.24(1.14)*** | 1.68(0.70)* | 1.80(0.79)* | 3.34(0.86)*** |
|  | *p = 0.008* | *p = 0.006* | *p < 0.001* | *p = 0.02* | *p = 0.03* | *p < 0.001* |
| **Maternal Age** | — | 0.01(0.05) | 0.02(0.07) | — | 0.03(0.05) | 0.05(0.05) |
|  |  | *p = 0.87* | *p = 0.81* |  | *p = 0.55* | *p = 0.36* |
| **Time of amniocentesis** | — | — | -0.28(0.20) | — | — | -0.57(0.16)*** |
|  |  |  | *p = 0.17* |  |  | *p < 0.001* |
| **p* < 0.05   ***p* < 0.01  ****p* < 0.001 (uncorrected)  Bonferroni significance threshold: *p* < 0.01; pT = Prenatal testosterone | | | | | | |

**Table S2 (continued).**

|  | Attention Switching | | | Attention to Detail | | | Imagination | | | |
| --- | --- | --- | --- | --- | --- | --- | --- | --- | --- | --- |
|  | M1  Baseline model | M2  M1 + maternal age | M3  M2 + time of amniocentesis | M1  Baseline model | M2  M1 + maternal age | M3  M2 + time of amniocentesis | M1  Baseline model | M2  M1 + maternal age | M3  M2 + time of amniocentesis |  |
| **pT** | -0.51(0.45) | -0.55(0.49) | -0.12(1.21) | -0.85(0.53) | -0.65(0.58) | -2.10(1.34) | -0.05(0.40) | -0.21(0.48) | -0.37(1.10) |  |
|  | *p = 0.26* | *p = 0.27* | *p = 0.93* | *p = 0.12* | *p = 0.27* | *p = 0.12* | *p = 0.90* | *p = 0.67* | *p = 0.74* |  |
| **Sex** | 1.73(0.78)* | 1.70(0.83) | 0.74(2.26) | 1.16(0.92) | 0.41(0.98) | 3.02(2.49) | 1.43(0.70) | 1.68(0.82)* | 1.90(2.05) |  |
|  | *p = 0.03* | *p = 0.05* | *p = 0.75* | *p = 0.21* | *p = 0.68* | *p = 0.23* | *p = 0.05* | *p = 0.04* | *p = 0.36* |  |
| **pT *x* Sex** | 0.34(0.90) | 0.59(0.95) | 0.13(2.43) | 0.86(1.07) | 0.29(1.12) | 3.35(2.68) | -0.11(0.81) | 0.20(0.93) | 0.62(2.20) |  |
|  | *p = 0.71* | *p = 0.54* | *p = 0.96* | *p = 0.43* | *p = 0.80* | *p = 0.22* | *p = 0.90* | *p = 0.84* | *p = 0.78* |  |
| **Rater** | -1.31(0.28)*** | -1.25(0.28)*** | -1.21(0.35)*** | -1.07(0.28)*** | -1.10(0.31)*** | -0.94(0.38)* | -0.50(0.24) | -0.46(0.26) | -0.26(0.33) |  |
|  | *p < 0.001* | *p < 0.001* | *p = 0.001* | *p < 0.001* | *p < 0.001* | *p = 0.02* | *p = 0.05* | *p = 0.08* | *p = 0.43* |  |
| **Pubertal Timing** | 0.01(0.55) | -0.06(0.57) | -0.63(0.76) | -0.14(0.65) | -0.42(0.66) | -0.26(0.84) | 0.27(0.49) | 0.31(0.55) | -0.12(0.69) |  |
|  | *p = 0.99* | *p = 0.91* | *p = 0.41* | *p = 0.83* | *p = 0.53* | *p = 0.76* | *p = 0.59* | *p = 0.58* | *p = 0.86* |  |
| **pT *x* Pubertal Timing** | 1.29(0.66) | 1.68(0.70)* | 1.93(0.95) | -0.32(0.79) | 0.14(0.83) | 0.23(1.05) | 1.11(0.59) | 1.14(0.69) | 1.41(0.85) |  |
|  | *p = 0.06* | *p = 0.02* | *p = 0.05* | *p = 0.69* | *p = 0.87* | *p = 0.83* | *p = 0.07* | *p = 0.10* | *p = 0.10* |  |
| **Maternal Age** | — | 0.01(0.04) | -0.03(0.06) | — | -0.01(0.05) | -0.06(0.06) | — | 0.004(0.04) | -0.03(0.05) |  |
|  |  | *p = 0.87* | *p = 0.64* |  | *p = 0.86* | *p = 0.35* |  | *p = 0.93* | *p = 0.55* |  |
| **Time of amniocentesis** | — | — | -0.39(0.17)* | — | — | -0.52(0.19)* | — | — | -0.25(0.15) |  |
|  |  |  | *p = 0.03* |  |  | *p = 0.01* |  |  | *p = 0.10* |  |
| **p* < 0.05   ***p* < 0.01  ****p* < 0.001 (uncorrected)  Bonferroni significance threshold: *p* < 0.01; pT = Prenatal testosterone | | | | | | | | | | |

**Figure S1.** Plotted pT *x* pubertal timing interaction for AQ subscales of social skill and communication, for males and females separately (from linear mixed model M2).


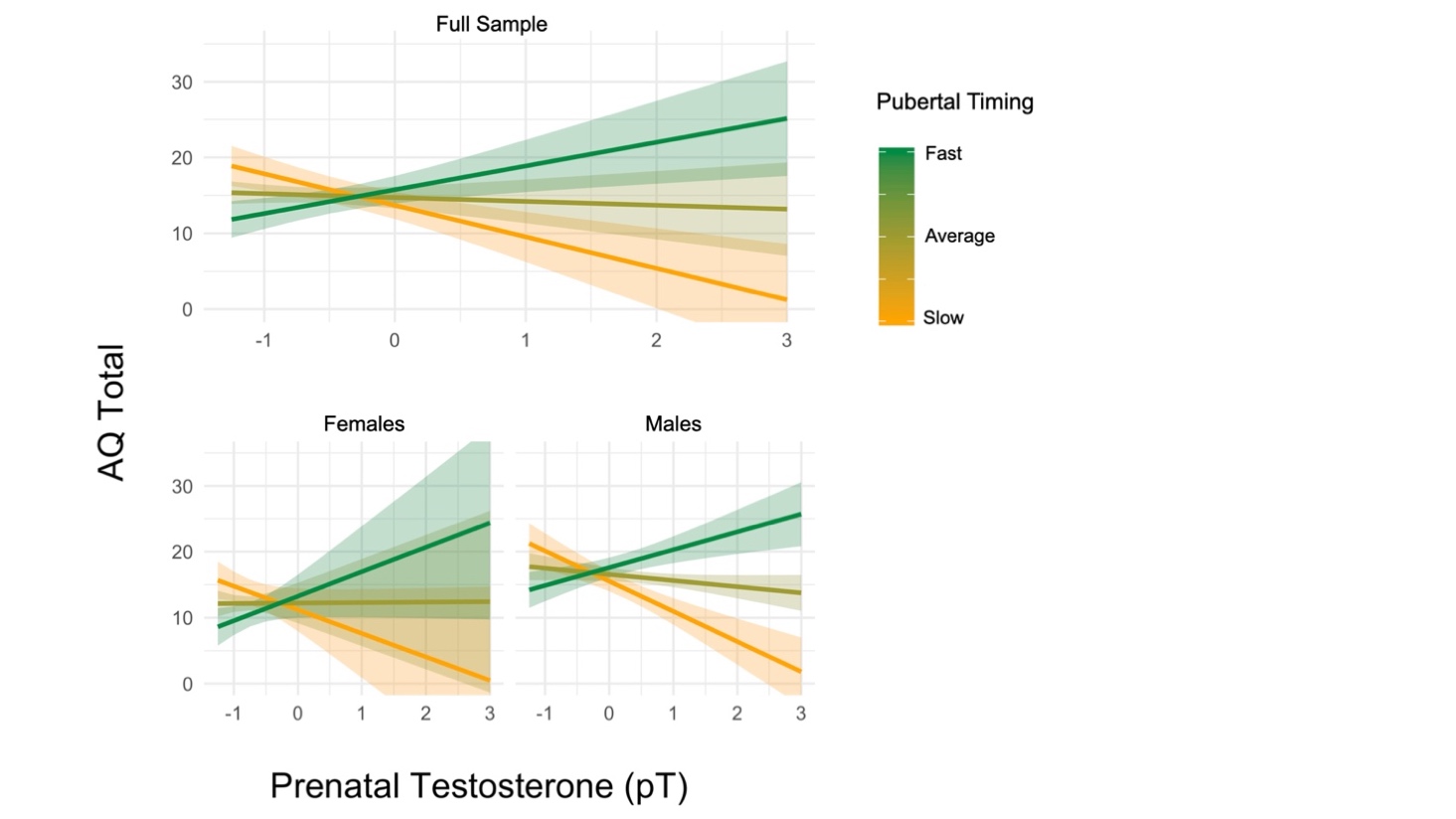

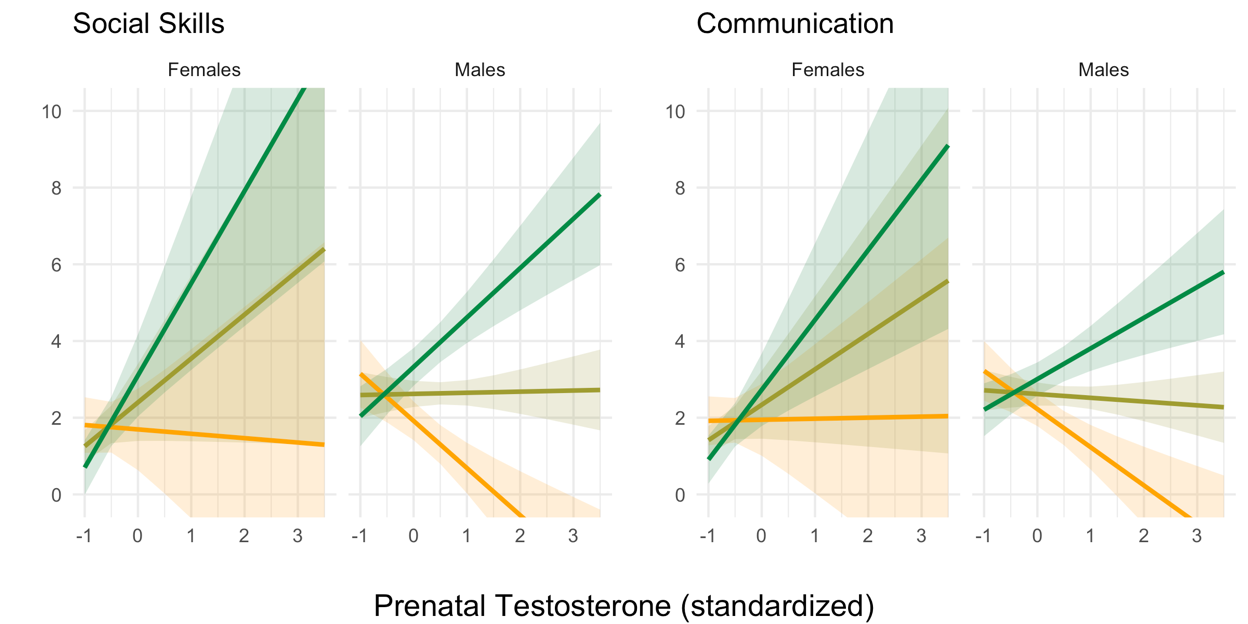


Sensitivity Analyses

### Sensitivity Analysis 1: Simpler models predicting AQ total

Table 2 in the main text shows that there was no main effect of pT on AQ totals when controlling for sex, pubertal measures, rater of the AQ (parent/self) and 2 pT interactions (pT-by-pubertal measures, pT-by-sex). We ran several simpler models to check whether a pT-AQ association existed in the context of less covariates, specifically without pubertal variables. A linear mixed model was run with just pT and sex as fixed effects (model A), the interaction between pT and sex was added in model B, and a fixed effect of rater (parent vs child) was added in model C. Across models A-C, no main effect of pT on AQ total was observed. Neither did they show any significant interaction between pT and sex (Table S3).

**Table S3.** Simpler linear mixed models predicting AQ total from pT, with just 1, 2 or 3 other covariates (model A, B and C, respectively).

|  | Effects on AQ total | | | |
| --- | --- | --- | --- | --- |
|  | Model A | Model B | | Model C |
| pT | -1.01(0.95) | | -0.98(1.83) | -0.97(1.86) |
|  | *p = 0.29* | | *p = 0.60* | *p = 0.61* |
| Sex | 6.20(1.82)*** | | 6.14(3.17) | 6.42(3.21) |
|  | *p = 0.001* | | *p = 0.06* | *p = 0.05* |
| pT *x* Sex |  | | -0.08(3.66) | -0.09(3.71) |
|  |  | | *p = 0.99* | *p = 0.99* |
| Rater |  | |  | -3.45(0.83)*** |
|  |  | |  | *p<0.001* |
| Observations | 171 | | 171 | 171 |
| Log Likelihood | -574.69 | | -572.47 | -563.98 |
| AIC | 1159.37 | | 1156.95 | 1141.96 |
| BIC | 1175.08 | | 1175.80 | 1163.95 |
| AIC = Akaike Information Criterion; BIC = Bayesian Information Criterion; pT = Prenatal testosterone; **p* < 0.05   ***p* < 0.01  ****p* < 0.001 (uncorrected) | | | | |

We also checked whether running separate linear models for self- and parent- report AQ total scores unmasked a main effect of pT. These results show that pT did not have any main effect on self or parent-report AQ total scores when these were analyzed separately (Tables S4-5).

**Table S4.** Predicting parent-report AQ total. Results of linear models (rather than linear mixed models).

|  |  | Model with pubertal stage | | |  | Model with pubertal timing | | | |
| --- | --- | --- | --- | --- | --- | --- | --- | --- | --- |
|  |  | M1  Baseline model | M2  M1 + maternal age | M3  M2 + time of amniocentesis |  | M1  Baseline model | M2  M1 + maternal age | M3  M2 + time of amniocentesis | |
| **M1** | pT | -0.68(2.23) | -1.15(2.55) | -3.77(5.93) |  | -0.85(2.21) | -1.25(2.53) | -3.95(5.83) | |
|  |  | *p = 0.77* | *p = 0.66* | *p = 0.53* |  | *p = 0.70* | *p = 0.63* | *p = 0.51* | |
|  | Sex | 8.71(4.08)* | 8.76(4.52) | 11.22(11.71) |  | 7.26(3.84) | 7.52(4.27) | 11.90(10.76) | |
|  |  | *p = 0.04* | *p = 0.06* | *p = 0.35* |  | *p = 0.07* | *p = 0.09* | *p = 0.28* | |
|  | pT *x* Sex | 1.54(4.63) | 2.61(5.15) | 10.51(11.64) |  | -0.76(4.43) | -0.06(4.90) | 6.76(11.70) | |
|  |  | *p = 0.74* | *p = 0.62* | *p = 0.38* |  | *p = 0.87* | *p = 1.00* | *p = 0.57* | |
|  | Pubertal Stage | 2.81(2.34) | 2.21(2.59) | -1.11(4.03) |  | -- | -- | -- | |
|  |  | *p = 0.24* | *p = 0.40* | *p = 0.79* |  |  |  |  | |
|  | pT *x* Pubertal Stage | 4.36(3.06) | 4.87(3.45) | 6.64(5.09) |  | -- | -- | -- | |
|  |  | *p = 0.16* | *p = 0.17* | *p = 0.20* |  |  |  |  | |
|  | Pubertal Timing | -- | -- | -- |  | 3.70(2.60) | 2.83(2.91) | -0.46(4.14) | |
|  |  |  |  |  |  | *p = 0.16* | *p = 0.34* | *p = 0.92* | |
|  | pT *x* Pubertal Timing | -- | -- | -- |  | 5.68(3.21) | 6.30(3.66) | 7.77(5.12) | |
|  |  |  |  |  |  | *p = 0.09* | *p = 0.10* | *p = 0.14* | |
| **M2** | Maternal Age at Birth | -- | -0.04(0.21) | -0.25(0.30) |  | -- | 0.01(0.21) | -0.20(0.30) | |
|  |  |  | *p = 0.84* | *p = 0.42* |  |  | *p = 0.98* | *p = 0.51* | |
| **M3** | Time of Amniocentesis | -- | -- | -1.93(0.93) |  | -- | -- | -1.91(0.91) | |
|  |  |  |  | *p = 0.05* |  |  |  | *p = 0.05* | |
|  | # Observations | 84 | 73 | 49 |  | 84 | 73 | 49 | |
|  | # Participants | 84 | 73 | 49 |  | 84 | 73 | 49 | |
|  | Adjusted R^2^ | 0.15 | 0.10 | 0.08 |  | 0.17 | 0.12 | 0.10 | |
| pT = Prenatal testosterone; AIC = Akaike Information Criterion; BIC = Bayesian Information Criterion.  **p* < 0.05   ***p* < 0.01  ****p* < 0.001 (uncorrected); Bonferroni-adjusted *p*-threshold = .0167 | | | | | | | | |  |

**Table S5.** Predicting adolescent self-report AQ total. Results of linear models (rather than linear mixed models)

|  |  | Model with pubertal stage | | |  | Model with pubertal timing | | |  |
| --- | --- | --- | --- | --- | --- | --- | --- | --- | --- |
|  |  | M1  Baseline model | M2  M1 + maternal age | M3  M2 + time of amniocentesis |  | M1  Baseline model | M2  M1 + maternal age | M3  M2 + time of amniocentesis |  |
| **M1** | pT | -0.89(2.00) | 0.89(2.05) | 4.93(5.36) |  | -0.95(1.97) | 0.79(2.01) | 5.33(5.20) |  |
|  |  | *p = 0.66* | *p = 0.67* | *p = 0. 37* |  | *p = 0.64* | *p = 0.70* | *p = 0.32* |  |
|  | Sex | 4.86(3.61) | 0.47(3.63) | -8.85(10.46) |  | 4.13(3.43) | 0.33(3.41) | -9.17(9.90) |  |
|  |  | *p = 0.19* | *p = 0.90* | *p = 0.41* |  | *p = 0.24* | *p = 0.93* | *p = 0.37* |  |
|  | pT *x* Sex | 0.94(4.17) | 0.85(4.12) | -0.92(10.61) |  | -1.09(3.95) | -2.66(3.86) | -10.36(10.45) |  |
|  |  | *p = 0.83* | *p = 0.84* | *p = 0.94* |  | *p = 0.79* | *p = 0.50* | *p = 0.33* |  |
|  | Pubertal Stage | 1.58(2.05) | 0.29(2.15) | -1.87(2.43) |  | -- | -- | -- |  |
|  |  | *p = 0.45* | *p = 0.90* | *p = 0.45* |  |  |  |  |  |
|  | pT *x* Pubertal Stage | 3.51(2.74) | 6.11(2.66)* | 14.14(3.06)*** |  | -- | -- | -- |  |
|  |  | *p = 0.21* | *p = 0.03* | *p < 0.001* |  |  |  |  |  |
|  | Pubertal Timing | -- | -- | -- |  | 0.43(2.49) | 0.83(2.30) | -1.64(2.42) |  |
|  |  |  |  |  |  | *p = 0.87* | *p = 0.72* | *p = 0.51* |  |
|  | pT *x* Pubertal Timing | -- | -- | -- |  | 6.04(2.94) | 8.00(2.79)* | 15.18(3.01)*** |  |
|  |  |  |  |  |  | *p = 0.05* | *p = 0.01* | *p < 0.001* |  |
| **M2** | Maternal Age at Birth | -- | 0.04(0.17) | 0.05(0.19) |  | -- | 0.09(0.17) | 0.13(0.18) |  |
|  |  |  | *p = 0.80* | *p = 0.81* |  |  | *p = 0.59* | *p = 0.49* |  |
| **M3** | Time of Amniocentesis | -- | -- | -2.37(0.53)*** |  | -- | -- | -2.29(0.44)*** |  |
|  |  |  |  | *p < 0.001* |  |  |  | *p < 0.001* |  |
|  | # Observations | 86 | 66 | 45 |  | 86 | 66 | 45 |  |
|  | # Participants | 86 | 66 | 45 |  | 86 | 66 | 45 |  |
|  | Adjusted R^2^ | 0.03 | 0.01 | 0.40 |  | 0.05 | 0.05 | 0.44 |  |
| pT = Prenatal testosterone; AIC = Akaike Information Criterion; BIC = Bayesian Information Criterion.  **p* < 0.05   ***p* < 0.01  ****p* < 0.001 (uncorrected); Bonferroni-adjusted *p*-threshold = .0167 | | | | | | | | | |

### Sensitivity Analysis 2: Sex-stratified models

In Figure 3, we split the interaction effect of pT-by-pubertal timing by sex which showed it was more reliable in males compared to females. However sex-stratified models were required to support this finding. Sex-stratified models also help support the lack of pT *x* sex interaction found in the linear mixed model. They are required as the absence of a 3-way interaction, pT *x* pubertal timing *x* sex in the model constrains male and female FT-AQ slope variations across the levels of pubertal timing to be equal (note in Figure 3 how the pT-AQ slopes vary between males and females but the *difference* in slopes imposed by the 3 levels of pubertal timing stays the same). We chose to run sex-stratified models instead of including a 3-way interaction given that these would also circumnavigate the potential for collinearity between pT and sex in the full-sample model. The sex-stratified models predicting AQ score included fixed effects of pT, rater, pubertal timing, pT-by-pubertal timing interaction in the baseline model (M1) and additionally maternal age (M2) and time of amniocentesis (M3) in sub-samples.

Results of the sex-stratified models verify that the interaction between pT and pubertal timing was stronger and more reliable in males compared to females (Tables S6-7, Figure S2). The effect in males did not reach significance by conventional standards in the baseline model (M1: *β =* 9.06, *SE* = 5.00, *p* = 0.07; n=55). In the fully-adjusted model, controlling for maternal age and time of amniocentesis, there was a significant interaction between pT and pubertal timing on AQ total in males (M3: *β =* 15.44, *SE* = 4.69, *p* = 0.001), however we interpret this effect with great caution due to the number of individuals (n=35) and observations (67) in this male-only test.

There was no significant main effect of pT on AQ total in either the male or female model.

**Table S6**. Predicting AQ total in **males** only. Estimates show *β (SE)* and *p*-values derived from Wald tests from linear mixed models.

|  |  | | Model with pubertal stage | | |  | | Model with pubertal timing | | |  |  |  |
| --- | --- | --- | --- | --- | --- | --- | --- | --- | --- | --- | --- | --- | --- |
|  |  | M1  Baseline model | | M2  M1 + maternal age | M3  M2 + time of amniocentesis | |  | M1  Baseline model | M2  M1 + maternal age | M3  M2 + time of amniocentesis | |  |  |
| **M1** | pT | -0.96(1.20) | | -0.43(1.28) | 0.34(1.32) | |  | -1.54(1.17) | -0.95(1.29) | -0.89(1.39) | |  |  |
|  |  | *p = 0.43* | | *p = 0.74* | *p = 0.80* | |  | *p = 0.19* | *p = 0.46* | *p = 0.53* | |  |  |
|  | Rater | -1.78(1.24) | | -1.67(1.37) | -1.76(1.55) | |  | -1.96(1.25) | -1.71(1.37) | -1.80(1.55) | |  |  |
|  |  | *p = 0.16* | | *p = 0.23* | *p = 0.26* | |  | *p = 0.12* | *p = 0.22* | *p = 0.25* | |  |  |
|  | Pubertal Stage | 3.94(3.16) | | -0.79(3.93) | -6.00(3.72) | |  | -- | -- | -- | |  |  |
|  |  | *p = 0.22* | | *p = 0.85* | *p = 0.11* | |  |  |  |  | |  |  |
|  | pT *x* Pubertal Stage | 2.39(4.69) | | 9.55(5.57) | 16.96(5.04)** | |  | -- | -- | -- | |  |  |
|  |  | *p = 0.62* | | *p = 0.09* | *p = 0.001* | |  |  |  |  | |  |  |
|  | Pubertal Timing | -- | | -- | -- | |  | -0.26(4.05) | 0.35(3.96) | -4.27(3.72) | |  |  |
|  |  |  | |  |  | |  | *p = 0.95* | *p = 0.93* | *p = 0.26* | |  |  |
|  | pT *x* Pubertal Timing | -- | | -- | -- | |  | 9.06(5.00) | 10.03(5.09) | 15.44(4.69)** | |  |  |
|  |  |  | |  |  | |  | *p = 0.07* | *p = 0.05* | *p = 0.001* | |  |  |
| **M2** | Maternal Age at Birth | -- | | 0.04(0.24) | -0.24(0.25) | |  | -- | 0.09(0.24) | -0.22(0.25) | |  |  |
|  |  |  | | *p = 0.87* | *p = 0.33* | |  |  | *p = 0.71* | *p = 0.40* | |  |  |
| **M3** | Time of Amniocentesis (gestational weeks) | -- | | -- | -2.65(0.69)*** | |  | -- | -- | -2.51(0.68)*** | |  |  |
|  |  |  | |  | *p < 0.001* | |  |  |  | *p < 0.001* | |  |  |
|  | # Observations | 96 | | 80 | 67 | |  | 96 | 80 | 67 | |  |  |
|  | # Participants | 55 | | 43 | 35 | |  | 55 | 43 | 35 | |  |  |
|  | Log Likelihood | -323.58 | | -265.57 | -214.37 | |  | -322.70 | -264.92 | -214.50 | |  |  |
|  | AIC | 661.15 | | 547.14 | 446.75 | |  | 659.40 | 545.83 | 447.00 | |  |  |
|  | BIC | 679.10 | | 566.19 | 466.59 | |  | 677.35 | 564.89 | 466.85 | |  |  |
| pT = Prenatal testosterone; AIC = Akaike Information Criterion; BIC = Bayesian Information Criterion.  **p* < 0.05   ***p* < 0.01  ****p* < 0.001 (uncorrected); Bonferroni-adjusted *p*-threshold = .0167 | | | | | | | | | | | | |  |

**Table S7**. Predicting AQ total in **females** only. Estimates show *β (SE)* and *p*-values derived from Wald tests from linear mixed models. Note: M3 coefficients should be interpreted with caution due to small number of females in this analysis.

|  |  | Model with pubertal stage | | |  | Model with pubertal timing | | |  |  |
| --- | --- | --- | --- | --- | --- | --- | --- | --- | --- | --- |
|  |  | M1  Baseline model | M2  M1 + maternal age | M3  M2 + time of amniocentesis |  | M1  Baseline model | M2  M1 + maternal age | M3  M2 + time of amniocentesis |  |  |
| **M1** | pT | -1.87(2.38) | -2.41(3.16) | 7.06(7.06) |  | -1.05(2.30) | -1.01(2.67) | 10.41(7.21) |  |  |
|  |  | *p = 0.44* | *p = 0.45* | *p = 0.32* |  | *p = 0.65* | *p = 0.71* | *p = 0.15* |  |  |
|  | Rater | -5.79(0.85)*** | -5.89(0.96)*** | -5.84(1.81)** |  | -5.73(0.86)*** | -5.91(0.96)*** | -5.86(1.71)*** |  |  |
|  |  | *p < 0.001* | *p < 0.001* | *p = 0.002* |  | *p < 0.001* | *p < 0.001* | *p < 0.001* |  |  |
|  | Pubertal Stage | -8.94(7.16) | -10.75(9.96) | -43.99(21.71) |  | -- | -- | -- |  |  |
|  |  | *p = 0.22* | *p = 0.29* | *p = 0.05* |  |  |  |  |  |  |
|  | pT *x* Pubertal Stage | -8.48(8.31) | -10.00(11.16) | -34.16(21.61) |  | -- | -- | -- |  |  |
|  |  | *p = 0.31* | *p = 0.38* | *p = 0.12* |  |  |  |  |  |  |
|  | Pubertal Timing | -- | -- | -- |  | -2.98(10.41) | -10.29(11.83) | -52.30(22.24)* |  |  |
|  |  |  |  |  |  | *p = 0.78* | *p = 0.39* | *p = 0.02* |  |  |
|  | pT *x* Pubertal Timing | -- | -- | -- |  | -2.30(12.13) | -9.03(13.55) | -39.93(22.06) |  |  |
|  |  |  |  |  |  | *p = 0.85* | *p = 0.51* | *p = 0.08* |  |  |
| **M2** | Maternal Age at Birth | -- | -0.13(0.18) | 0.36(0.26) |  | -- | -0.12(0.18) | 0.60(0.27)* |  |  |
|  |  |  | *p = 0.47* | *p = 0.17* |  |  | *p = 0.51* | *p = 0.03* |  |  |
| **M3** | Time of Amniocentesis (gestational weeks) | -- | -- | 0.88(1.18) |  | -- | -- | 1.25(1.13) |  |  |
|  |  |  |  | *p = 0.46* |  |  |  | *p = 0.27* |  |  |
|  | # Observations | 74 | 59 | 27 |  | 74 | 59 | 27 |  |  |
|  | # Participants | 40 | 30 | 14 |  | 40 | 30 | 14 |  |  |
|  | Log Likelihood | -204.76 | -162.00 | -64.69 |  | -205.12 | -161.78 | -63.39 |  |  |
|  | AIC | 423.53 | 339.99 | 147.37 |  | 424.23 | 339.56 | 144.78 |  |  |
|  | BIC | 439.66 | 356.61 | 159.03 |  | 440.36 | 356.18 | 156.44 |  |  |
| pT = Prenatal testosterone; AIC = Akaike Information Criterion; BIC = Bayesian Information Criterion.  **p* < 0.05   ***p* < 0.01  ****p* < 0.001 (uncorrected); Bonferroni-adjusted *p*-threshold = .0167 | | | | | | | | | |  |

**Figure S2.** Interaction between pT and pubertal timing on AQ total, from sex-stratified analysis (M2). Unlike Figure 3, slope-differences between the 3 pubertal timing levels are allowed to differ between males and females. AQ values (y-axis) capture the average of parent- and self- reported AQ. Error bands refer to standard errors.


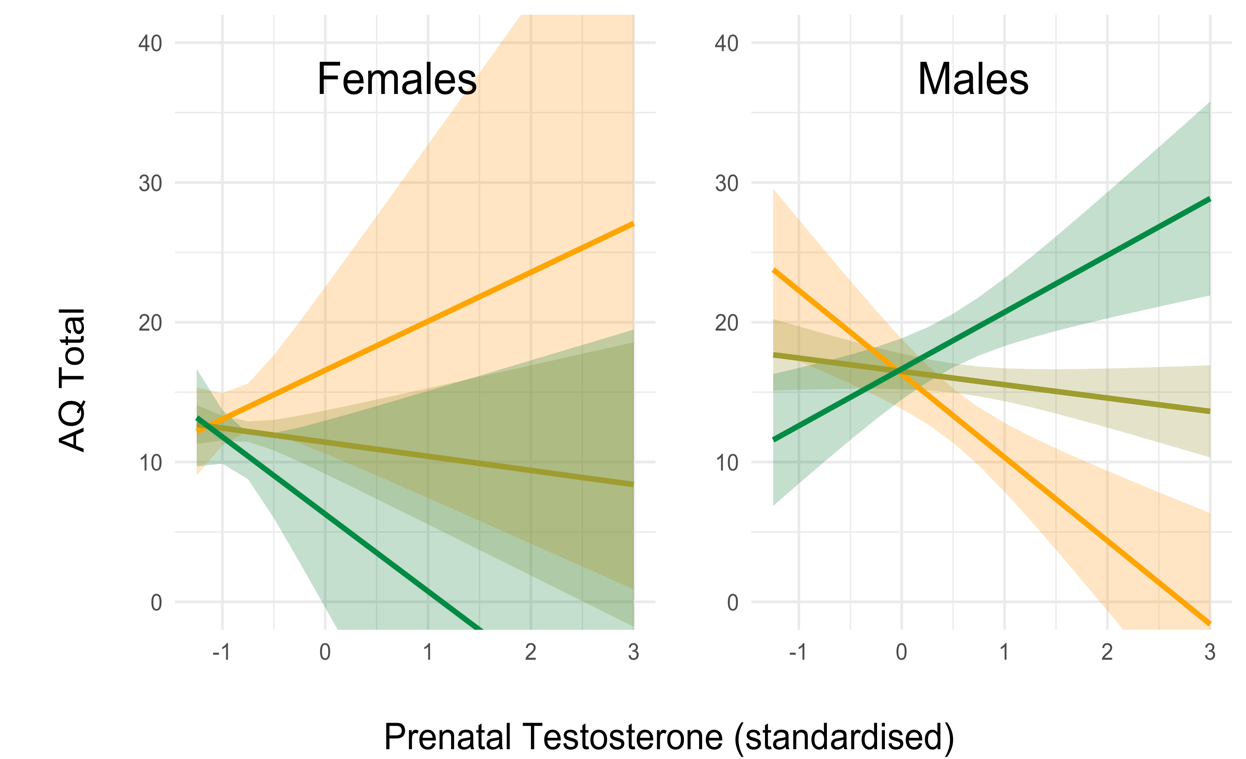

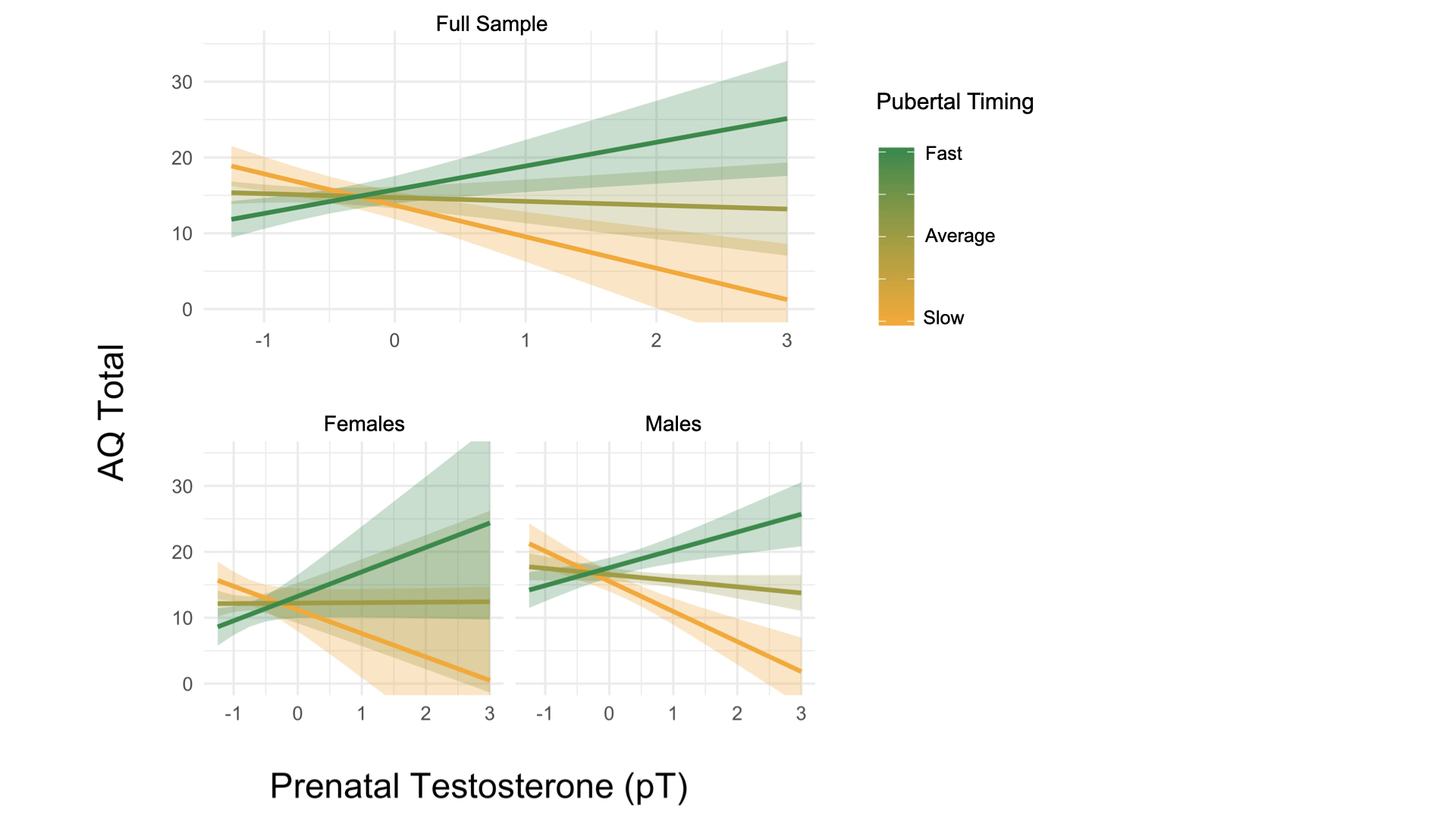


### Sensitivity Analysis 3: Generalized Linear Mixed Models

There was some non-normality and heteroskedasticity among the residuals of the linear mixed models predicting AQ total (i.e. models presented in Table 2). An example of this from M1 is shown in Figure S3 (top row). Neither log transformation of pT levels nor AQ total scores corrected this heteroskedasticity, but even if they did, transforming the outcome or primary predictor of interest would make interpretation of coefficients more abstract (e.g. 1 unit change in pT related to X point change in log(AQ)). Generalized linear models have been proposed as an alternative method to transforming the outcome (Ng and Cribbie, 2017). Standard general linear models (e.g. regression, ANOVA) assume that residuals are normally distributed and homoscedastic. They are comprised of (1) an “identity” link function that transforms the linear combination of predictors and their coefficients (i.e. error + B1*X1 + B2*X2…) directly to predicted outcome (Y’) and (2) an assumed normal distribution of errors. In generalized linear models, one can specify both the link function (e.g. log link, square root link) and the assumed distribution of errors (e.g. gaussian, gamma, Poisson). Using a generalized mixed model using the *glmer* function in R (lme4 package), we found that when assuming a gamma distribution and maintaining an identity link function, the residual spread was improved (see Figure S3, bottom row).

Results of these generalized linear mixed models (Table S8) supported the null relationship between pT and AQ totals suggested by linear mixed models: pT was not associated with AQ scores, neither in the full sample (non-significant main effect of pT), nor in one sex alone (non-significant pT-by-sex interaction). However the generalized linear mixed model did not support the interaction between pT and pubertal timing on AQ totals suggested by the linear mixed models. While this effect was significant at the corrected threshold in linear mixed models (e.g. M2: *β =*7.28, *SE* = 2.74, *t* = 2.65, *p*=0.01; *N*=73), effect size was reduced and non-significant in all generalized linear mixed models (e.g. M2: *β =*4.78, *SE* = 2.58, *t* = 1.85, *p*=0.06; *N*=73).

**Figure S3.** Model residuals from linear mixed models M1 (top row) which assumes a normally distributed errors, and residuals from generalized mixed models with an identity link and assuming a gamma distribution (bottom row).


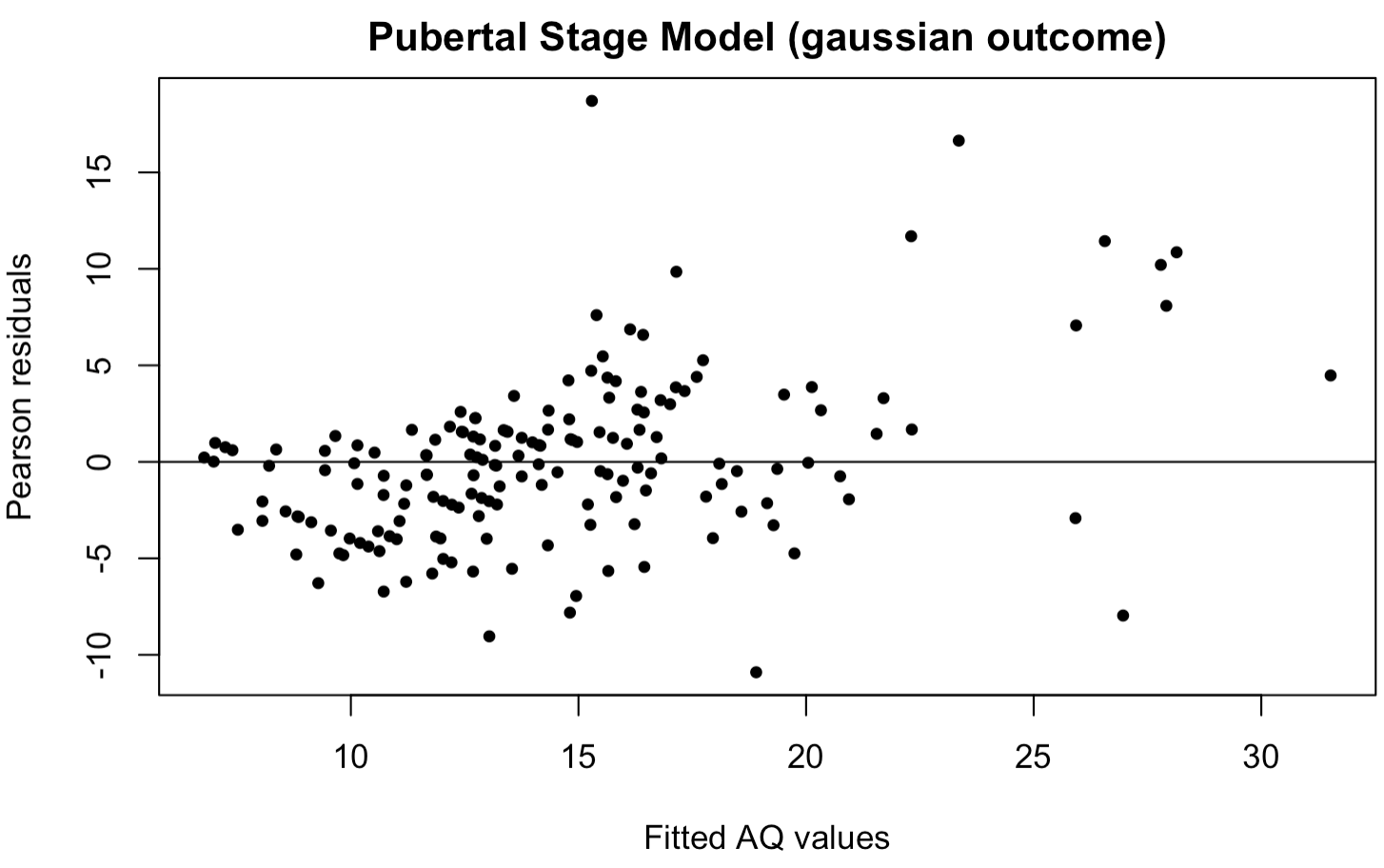

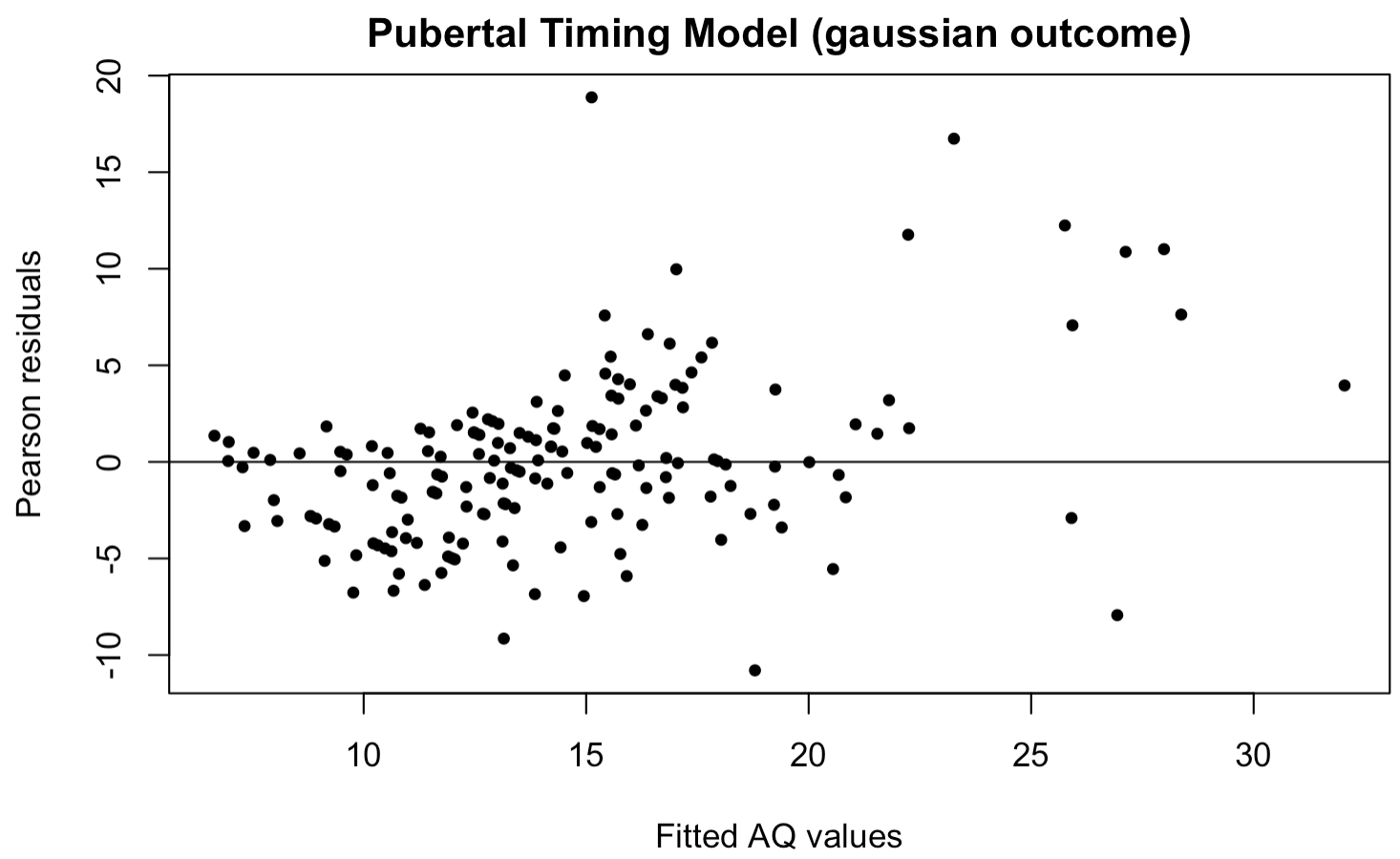


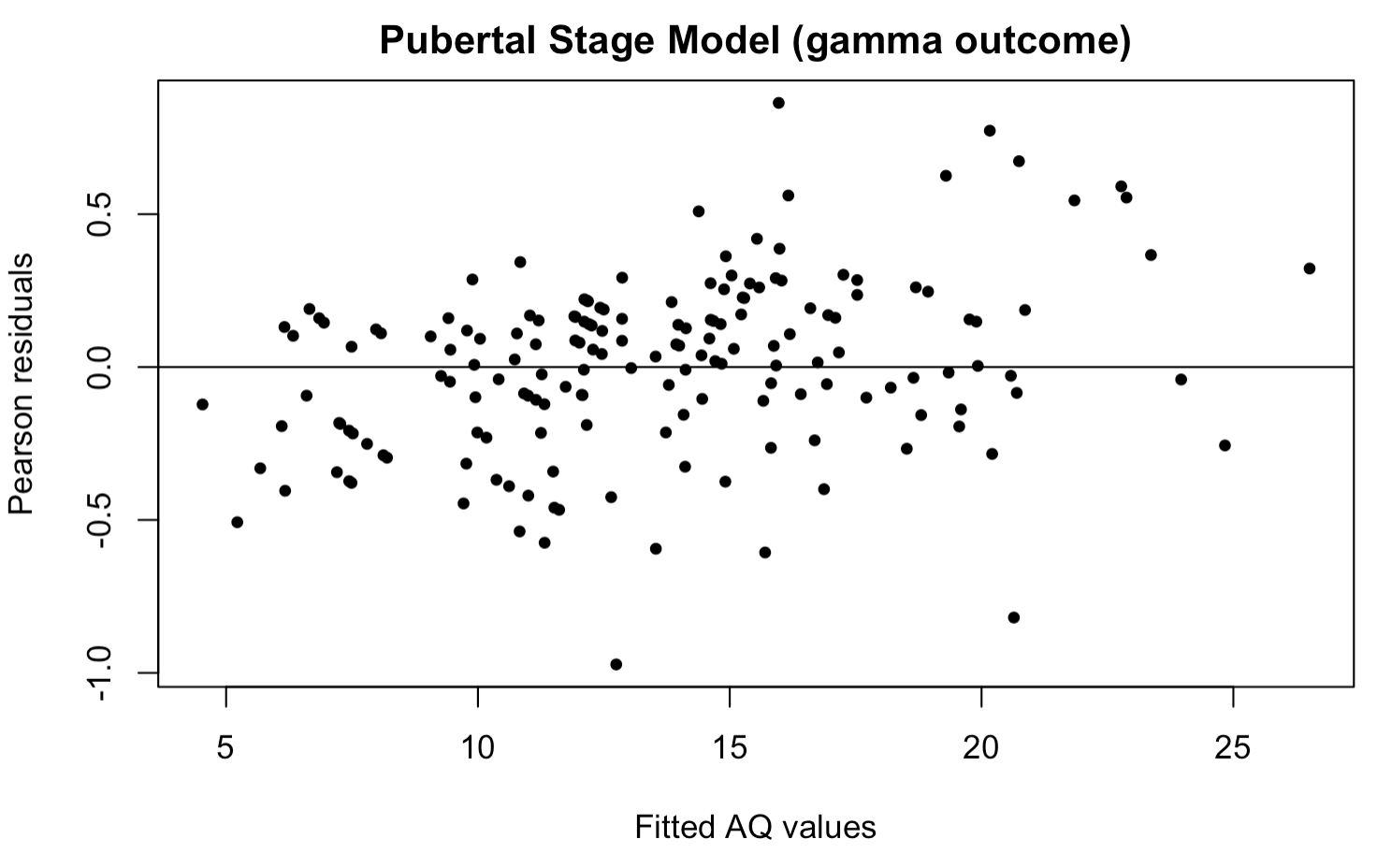

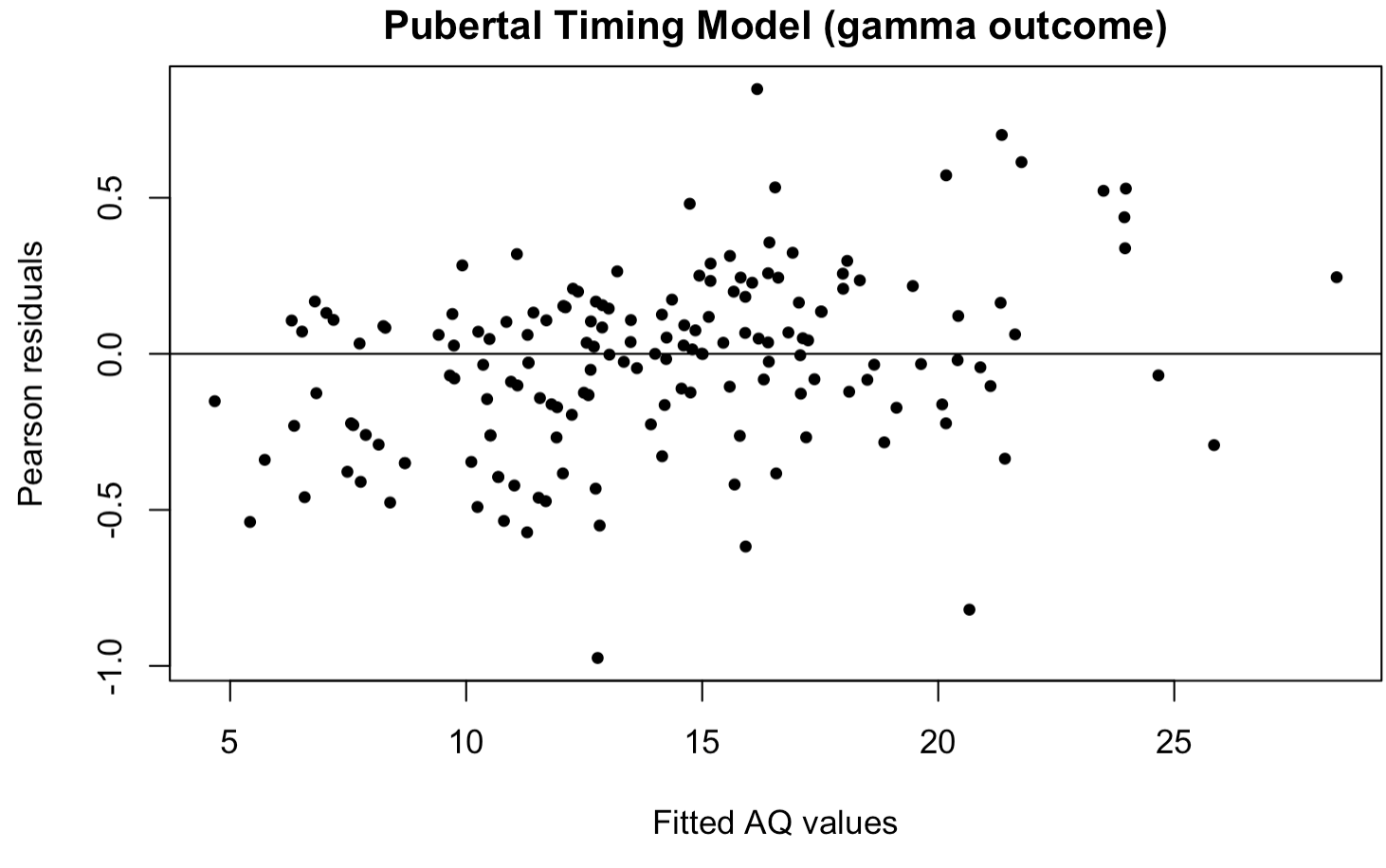


**Table S8.** Results of Generalized Linear Mixed Model (GLMM) assuming gamma distribution predicting AQ totals (as opposed to linear mixed model (LMM).

|  |  | Models with pubertal stage | | |  | Models with pubertal timing | | |  |  |
| --- | --- | --- | --- | --- | --- | --- | --- | --- | --- | --- |
|  |  | M1  Baseline model | M2  M1 + maternal age | M3  M2 + time of amniocentesis |  | M1  Baseline model | M2  M1 + maternal age | M3  M2 + time of amniocentesis |  |  |
| **M1** | pT | -0.91(1.52) | -0.83 (1.68) | -3.11(3.50) |  | -0.94(1.52) | -0.90(1.67) | -3.34(3.47) |  |  |
|  |  | *p = 0.55* | *p = 0.63* | *p = 0.38* |  | *p = 0.54* | *p = 0.60* | *p = 0.34* |  |  |
|  | Sex | 6.04(2.79)* | 4.84(3.02) | 7.33(6.97) |  | 5.41(2.61)* | 4.41 (2.82) | 8.40(6.35) |  |  |
|  |  | *p = 0.04* | *p = 0.11* | *p = 0.30* |  | *p = 0.04* | *p = 0.12* | *p = 0.19* |  |  |
|  | Rater | -4.67(0.69)*** | -4.71(0.77)*** | -4.01(0.98)*** |  | -4.66(0.68)*** | -4.70(0.77)*** | -4.05(0.97)*** |  |  |
|  |  | *p < 0.001* | *p < 0.001* | *p < .001* |  | *p < 0.001* | *p < 0.001* | *p < 0.001* |  |  |
|  | pT *x* Sex | 1.84(3.19) | 2.06(3.42) | 10.20(6.91) |  | 0.18(3.04) | 0.04(3.24) | 6.75(6.96) |  |  |
|  |  | *p = 0.57* | *p = 0.55* | *p = 0.14* |  | *p = 0.96* | *p = 0.99* | *p = 0.34* |  |  |
|  | Pubertal Stage | 1.20(1.64) | 0.78(1.80) | -1.51(2.69) |  | -- | -- | -- |  |  |
|  |  | *p = 0.47* | *p = 0.67* | *p = 0.58* |  |  |  |  |  |  |
|  | pT *x* Pubertal Stage | 3.02(2.21) | 3.63(2.36) | 6.30(3.43) |  | -- | -- | -- |  |  |
|  |  | *p = 0.18* | *p = 0.13* | *p = 0.07* |  |  |  |  |  |  |
|  | Pubertal Timing | -- | -- | -- |  | 1.73(1.99) | 1.07(2.06) | -1.01(2.84) |  |  |
|  |  |  |  |  |  | *p = 0.39* | *p = 0.61* | *p = 0.73* |  |  |
|  | pT *x* Pubertal Timing | -- | -- | -- |  | 3.91(2.44) | 4.78(2.58) | 6.96(3.54) |  |  |
|  |  |  |  |  |  | *p = 0.11* | *p = 0.06* | *p = 0.05* |  |  |
| **M2** | Maternal Age at Birth | -- | -0.04(0.14) | -0.11(0.19) |  | -- | -0.01(0.14) | -0.08(0.20) |  |  |
|  |  |  | *p = 0.78* | *p = 0.58* |  |  | *p = 0.93* | *p = 0.70* |  |  |
| **M3** | Time of Amniocentesis (gestational weeks) | -- | -- | -1.52(0.59)* |  | -- | -- | -1.51(0.58)* |  |  |
|  |  |  |  | *p = 0.01* |  |  |  | *p = 0.01* |  |  |
|  | # Observations | 170 | 139 | 94 |  | 170 | 139 | 94 |  |  |
|  | # Participants | 95 | 73 | 49 |  | 95 | 73 | 49 |  |  |
|  | Log Likelihood | -514.27 | -418.33 | -291.42 |  | -513.70 | -417.77 | -291.19 |  |  |
|  | AIC | 1046.53 | 856.66 | 604.84 |  | 1045.39 | 855.55 | 604.39 |  |  |
|  | BIC | 1074.76 | 886.01 | 632.81 |  | 1073.62 | 884.89 | 632.36 |  |  |
| pT = Prenatal testosterone; AIC = Akaike Information Criterion; BIC = Bayesian Information Criterion.  **p* < 0.05   ***p* < 0.01  ****p* < 0.001 (uncorrected); Bonferroni-adjusted *p*-threshold = .0167 | | | | | | | | | |  |

Following the same rationale, we used generalized linear mixed models to explore the effects of pT on the AQ sub-scales, in particular the robustness of the pT *x* pubertal timing interaction on the two subscales which showed effects in linear mixed models (social skills, communication). Distributions of AQ sub-scales were more skewed than AQ totals making this analysis an important addition to linear mixed model results for interpretation. Table S9 below shows that, like the linear mixed models, there was no significant main effect of pT on any subscale. The interaction between pT and pubertal timing on the AQ subscale of social skills was significant at the corrected alpha-threshold in all models (M1-M3). The interaction between pT and pubertal timing on communication became non-significant in general linear mixed models M1 and M2. The significant interaction of pT *x* pubertal timing on communication in M3 should be interpreted with caution given the small number of observations in this model (# observations = 94; *n* = 49).

**Table S9.** Results of Generalized Linear Mixed Models predicting AQ subscales Social Skills and Communication

|  | Social Skills | | | Communication | | |
| --- | --- | --- | --- | --- | --- | --- |
|  | M1  Baseline model | M2  M1 + maternal age | M3  M2 + time of amniocentesis | M1  Baseline model | M2  M1 + maternal age | M3  M2 + time of amniocentesis |
| **pT** | 0.32(0.38) | 0.57(0.47) | -0.13(0.83) | 0.08(0.34) | 0.36(0.41) | 1.30(0.78) |
|  | *p = 0.41* | *p = 0.23* | *p = 0.88* | *p = 0.82* | *p = 0.37* | *p = 0.10* |
| **Sex** | 0.66(0.68) | 0.07(0.80) | 1.03(1.53) | 0.94(0.62) | 0.39(0.72) | -1.33(1.46) |
|  | *p = 0.33* | *p = 0.93* | *p = 0.51* | *p = 0.13* | *p = 0.59* | *p = 0.37* |
| **pT *x* Sex** | -0.53(0.77) | -0.73(0.91) | 0.32(1.66) | -0.84(0.68) | -1.07(0.79) | -2.10(1.56) |
|  | *p = 0.49* | *p = 0.42* | *p = 0.85* | *p = 0.22* | *p = 0.18* | *p = 0.19* |
| **Rater** | -0.58(0.25) | -0.62(0.25) | -0.26(0.36) | -0.33(0.25) | -0.25(0.28) | -0.23(0.29) |
|  | *p = 0.02* | *p = 0.02* | *p = 0.47* | *p = 0.19* | *p = 0.39* | *p = 0.43* |
| **Pubertal Timing** | 1.25(0.51) | 1.26(0.57) | -0.61(0.61) | 0.07(0.44) | -0.21(0.48) | -0.13(0.56) |
|  | *p = 0.02* | *p = 0.03* | *p = 0.32* | *p = 0.87* | *p = 0.66* | *p = 0.81* |
| **pT *x* Pubertal Timing** | 1.92(0.60)** | 2.20(0.67)** | 3.52(0.74)*** | 0.88(0.52) | 0.90(0.58) | 2.09(0.72)** |
|  | *p = 0.002* | *p = 0.002* | *p < 0.001* | *p = 0.10* | *p = 0.13* | *p = 0.006* |
| **Maternal Age** | — | 0.005(0.04) | 0.07(0.05) | — | 0.004(0.03) | 0.03(0.04) |
|  |  | *p = 0.91* | *p = 0.17* |  | *p = 0.90* | *p = 0.40* |
| **Time of amniocentesis** | — | — | -0.07(0.16) | — | — | -0.55(0.10)*** |
|  |  |  | *p = 0.66* |  |  | *p < 0.001* |
| **p* < 0.05   ***p* < 0.01  ****p* < 0.001 (uncorrected)  Bonferroni significance threshold: *p* < 0.01; pT = Prenatal testosterone | | | | | | |

### Sensitivity Analysis 4: Excluding clinical cases of autism

There was just one autistic participant in the sample. This individual’s AQ score was reported by the parent only (no self-report available) thus removing their data from the analysis reduced the number of observations from 170 to 169. As expected, results remained highly similar after removing them. No main effect of pT was observed on AQ total or any AQ subscale. An interaction between pT and pubertal timing on AQ total remained (M1: *β* = 5.65, *SE* = 2.48, *t* = 2.28, *p* = 0.03; M2: *β* = 6.82, *SE* = 2.60, *t* = 2.63, *p* = 0.009; M3: *β* = 11.15, *SE* = 3.27, *t* =3.41, *p* = 0.001), as did the interactions between pT and pubertal timing on social skills (M1: *β =* 2.10, *SE* = 0.80, *t* = 2.64, *p* = 0.009; M2: *β =* 2.37, *SE* = 0.86, *t* = 2.77, *p* = 0.006; M3: *β =* 4.24, *SE* = 1.14, *t* = 3.72, *p* < 0.001).

**Table S10.** Items of the Adult AQ (completed by participants about themselves) and the Adolescent AQ (completed by parents about their children)

|  | **Adult AQ (Self-report; Baron-Cohen et al., 2001)** | **Adolescent AQ (Parent-report; Baron-Cohen et al., 2006)** |
| --- | --- | --- |
| 1 | I prefer to do things with others rather than on my own. | S/he prefers to do things with others rather than on her/his own. |
| 2 | I prefer to do things the same way over and over again. | S/he prefers to do things the same way over and over again. |
| 3 | If I try to imagine something, I find it very easy to create a picture in my mind. | If s/he tries to imagine something, s/he finds it very easy to create a picture in her/his mind. |
| 4 | I frequently get so strongly absorbed in one thing that I lose sight of other things. | S/he frequently gets so strongly absorbed in one thing that s/he loses sight of other things. |
| 5 | I often notice small sounds when others do not. | S/he often notices small sounds when others do not. |
| 6 | I usually notice car number plates or similar strings of information. | S/he usually notices car number plates or similar strings of information. |
| 7 | Other people frequently tell me that what I’ve said is impolite, even though I think it is polite. | Other people frequently tell her/him that what s/he has said is impolite, even though s/he thinks it is polite. |
| 8 | When I’m reading a story, I can easily imagine what the characters might look like. | When s/he is reading a story, s/he can easily imagine what the characters might look like. |
| 9 | I am fascinated by dates. | S/he is fascinated by dates. |
| 10 | In a social group, I can easily keep track of several different people’s conversations. | In a social group, s/he can easily keep track of several different people’s conversations. |
| 11 | I find social situations easy. | S/he finds social situations easy. |
| 12 | I tend to notice details that others do not. | S/he tends to notice details that others do not. |
| 13 | I would rather go to a library than a party. | S/he would rather go to a library than a party. |
| 14 | I find making up stories easy. | S/he finds making up stories easy. |
| 15 | I find myself drawn more strongly to people than to things. | S/he finds her/himself drawn more strongly to people than to things. |
| 16 | I tend to have very strong interests which I get upset about if I can’t pursue. | S/he tends to have very strong interests, which s/he gets upset about if s/he can’t pursue. |
| 17 | I enjoy social chit-chat. | S/he enjoys social chit-chat. |
| 18 | When I talk, it isn’t always easy for others to get a word in edgeways. | When s/he talks, it isn’t always easy for others to get a word in edgeways. |
| 19 | I am fascinated by numbers. | S/he is fascinated by numbers. |
| 20 | When I’m reading a story, I find it difficult to work out the characters’ intentions. | When s/he is reading a story, s/he finds it difficult to work out the characters’ intentions. |
| 21 | I don’t particularly enjoy reading fiction. | S/he doesn’t particularly enjoy reading fiction. |
| 22 | I find it hard to make new friends. | S/he finds it hard to make new friends. |
| 23 | I notice patterns in things all the time. | S/he notices patterns in things all the time. |
| 24 | I would rather go to the theatre than a museum. | S/he would rather go to the theatre than a museum. |
| 25 | It does not upset me if my daily routine is disturbed. | It does not upset him/her if his/her daily routine is disturbed. |
| 26 | I frequently find that I don’t know how to keep a conversation going. | S/he frequently finds that s/he doesn’t know how to keep a conversation going. |
| 27 | I find it easy to “read between the lines” when someone is talking to me. | S/he finds it easy to “read between the lines” when someone is talking to her/him. |
| 28 | I usually concentrate more on the whole picture, rather than the small details. | S/he usually concentrates more on the whole picture, rather than the small details. |
| 29 | I am not very good at remembering phone numbers. | S/he is not very good at remembering phone numbers. |
| 30 | I don’t usually notice small changes in a situation, or a person’s appearance. | S/he doesn’t usually notice small changes in a situation, or a person’s appearance. |
| 31 | I know how to tell if someone listening to me is getting bored. | S/he knows how to tell if someone listening to him/her is getting bored. |
| 32 | I find it easy to do more than one thing at once. | S/he finds it easy to do more than one thing at once. |
| 33 | When I talk on the phone, I’m not sure when it’s my turn to speak. | When s/he talks on the phone, s/he is not sure when it’s her/his turn to speak. |
| 34 | I enjoy doing things spontaneously. | S/he enjoys doing things spontaneously. |
| 35 | I am often the last to understand the point of a joke. | S/he is often the last to understand the point of a joke. |
| 36 | I find it easy to work out what someone is thinking or feeling just by looking at their face. | S/he finds it easy to work out what someone is thinking or feeling just by looking at their face. |
| 37 | If there is an interruption, I can switch back to what I was doing very quickly. | If there is an interruption, s/he can switch back to what s/he was doing very quickly. |
| 38 | I am good at social chit-chat. | S/he is good at social chit-chat. |
| 39 | People often tell me that I keep going on and on about the same thing. | People often tell her/him that s/he keeps going on and on about the same thing. |
| 40 | When I was young, I used to enjoy playing games involving pretending with other children. | When s/he was younger, s/he used to enjoy playing games involving pretending with other children. |
| 41 | I like to collect information about categories of things (e.g. types of car, types of bird, types of train, types of plant, etc.). | S/he likes to collect information about categories of things (e.g. types of car, types of bird, types of train, types of plant, etc.). |
| 42 | I find it difficult to imagine what it would be like to be someone else. | S/he finds it difficult to imagine what it would be like to be someone else. |
| 43 | I like to plan any activities I participate in carefully. | S/he likes to plan any activities s/he participates in carefully. |
| 44 | I enjoy social occasions. | S/he enjoys social occasions. |
| 45 | I find it difficult to work out people’s intentions. | S/he finds it difficult to work out people’s intentions. |
| 46 | New situations make me anxious. | New situations make him/her anxious. |
| 47 | I enjoy meeting new people. | S/he enjoys meeting new people. |
| 48 | I am a good diplomat. | S/he is a good diplomat. |
| 49 | I am not very good at remembering people’s date of birth. | S/he is not very good at remembering people’s date of birth. |
| 50 | I find it very easy to play games with children that involve pretending. | S/he finds it very easy to play games with children that involve pretending. |

**Figure S4.** Plot of how pubertal timing measure was created. Pubertal stage (y-axis) regressed on age (x-axis) for males and females separately. Size of residuals (vertical lines) reflected each individual’s pubertal timing (i.e. deviation from the mean pubertal stage for age).


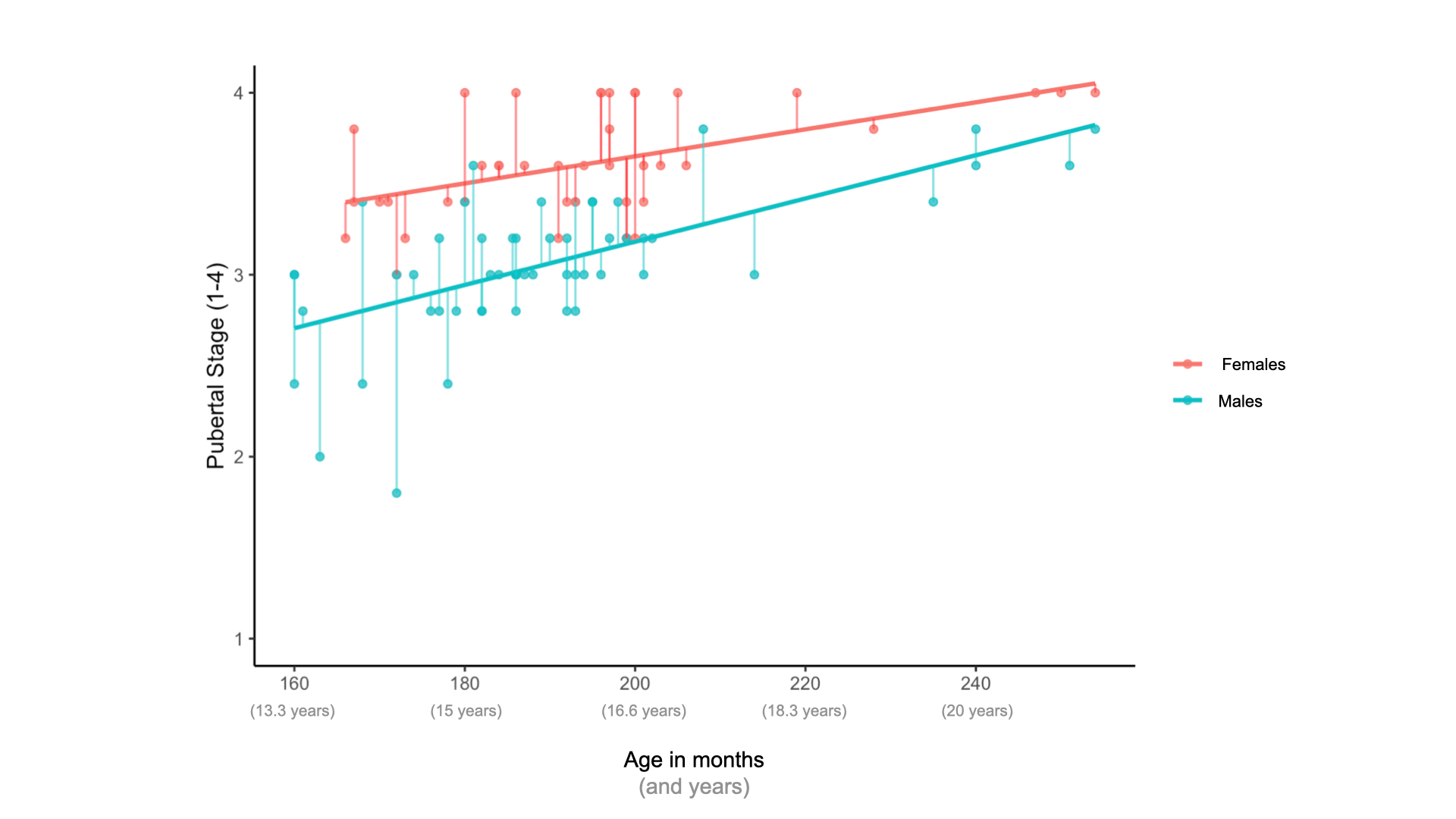


References

Abbott, D.H., Tarantal, A.F., Dumesic, D.A., 2009. Fetal, infant, adolescent and adult phenotypes of polycystic ovary syndrome in prenatally androgenized female rhesus monkeys. American Journal of Primatology: Official Journal of the American Society of Primatologists 71, 776-784.

Alaerts, K., Swinnen, S.P., Wenderoth, N., 2016. Sex differences in autism: a resting-state fMRI investigation of functional brain connectivity in males and females. Social Cognitive and Affective Neuroscience 11, 1002-1016.

Austin, E.J., 2005. Personality correlates of the broader autism phenotype as assessed by the Autism Spectrum Quotient (AQ). Personality and Individual Differences 38, 451-460.

Auyeung, B., Ahluwalia, J., Thomson, L., Taylor, K., Hackett, G., O’Donnell, K.J., Baron-Cohen, S., 2012. Prenatal versus postnatal sex steroid hormone effects on autistic traits in children at 18 to 24 months of age. Molecular autism 3, 17.

Auyeung, B., Baron-Cohen, S., Ashwin, E., Knickmeyer, R., Hackett, G., Hines, M., 2009a. Fetal Testosterone Predicts Sexually Differentiated Childhood Behaviour in Girls and Boys. Psychological Science.

Auyeung, B., Baron-Cohen, S., Ashwin, E., Knickmeyer, R., Taylor, K., Hackett, G., 2009b. Fetal testosterone and autistic traits. Br J Psychol 100, 1-22.

Auyeung, B., Baron-Cohen, S., Chapman, E., Knickmeyer, R., Taylor, K., Hackett, G., 2006. Foetal testosterone and the child systemizing quotient. European Journal of Endocrinology 155, S123-S130.

Auyeung, B., Taylor, K., Hackett, G., Baron-Cohen, S., 2010. Foetal testosterone and autistic traits in 18 to 24-month-old children. Molecular autism 1, 11.

Baron-Cohen, S., Auyeung, B., Nørgaard-Pedersen, B., Hougaard, D.M., Abdallah, M.W., Melgaard, L., Cohen, A.S., Chakrabarti, B., Ruta, L., Lombardo, M.V., 2015. Elevated fetal steroidogenic activity in autism. Molecular Psychiatry 20, 369.

Baron-Cohen, S., Hoekstra, R.A., Knickmeyer, R., Wheelwright, S., 2006. The autism-spectrum quotient (AQ)—adolescent version. Journal of autism and developmental disorders 36, 343.

Baron-Cohen, S., Lombardo, M.V., Auyeung, B., Ashwin, E., Chakrabarti, B., Knickmeyer, R., 2011. Why are autism spectrum conditions more prevalent in males? PLoS Biology 9, e1001081.

Baron-Cohen, S., Lutchmaya, S., Knickmeyer, R., 2004. Prenatal testosterone in mind: Studies of amniotic fluid. MIT Press, Cambridge, MA.

Baron-Cohen, S., Tsompanidis, A., Auyeung, B., Nørgaard-Pedersen, B., Hougaard, D.M., Abdallah, M., Cohen, A., Pohl, A., 2019. Foetal oestrogens and autism. Molecular psychiatry, 1-9.

Baron-Cohen, S., Wheelwright, S., Skinner, R., Martin, J., Clubley, E., 2001. The autism-spectrum quotient (AQ): Evidence from asperger syndrome/high-functioning autism, malesand females, scientists and mathematicians. Journal of autism and developmental disorders 31, 5-17.

Barry, J.A., Hardiman, P.J., Siddiqui, M., Thomas, M., 2011. Meta-analysis of sex difference in testosterone levels in umbilical cord blood. Journal of obstetrics and gynaecology 31, 697-702.

Bates, D., Mächler, M., Bolker, B., Walker, S., 2014. Fitting linear mixed-effects models using lme4. arXiv preprint arXiv:1406.5823.

Bejerot, S., Eriksson, J.M., Bonde, S., Carlström, K., Humble, M.B., Eriksson, E., 2012. The extreme male brain revisited: gender coherence in adults with autism spectrum disorder. The British Journal of Psychiatry 201, 116-123.

Beking, T., Geuze, R.H., van Faassen, M., Kema, I.P., Kreukels, B.P.C., Groothuis, T.G.G., 2018. Prenatal and pubertal testosterone affect brain lateralization. Psychoneuroendocrinology 88, 78-91.

Berni, T.R., Morgan, C.L., Berni, E.R., Rees, D.A., 2018. Polycystic Ovary Syndrome Is Associated With Adverse Mental Health and Neurodevelopmental Outcomes. J Clin Endocrinol Metab 103, 2116-2125.

Billstedt, E., Gillberg, I.C., Gillberg, C., 2005. Autism after adolescence: population-based 13- to 22-year follow-up study of 120 individuals with autism diagnosed in childhood. J Autism Dev Disord 35, 351-360.

Blakemore, S.-J., 2012. Development of the social brain in adolescence. Journal of the Royal Society of Medicine 105, 111-116.

Bond, L., Clements, J., Bertalli, N., Evans-Whipp, T., McMorris, B.J., Patton, G.C., Toumbourou, J.W., Catalano, R.F., 2006. A comparison of self-reported puberty using the Pubertal Development Scale and the Sexual Maturation Scale in a school-based epidemiologic survey. Journal of adolescence 29, 709-720.

Braams, B.R., van Duijvenvoorde, A.C., Peper, J.S., Crone, E.A., 2015. Longitudinal changes in adolescent risk-taking: a comprehensive study of neural responses to rewards, pubertal development, and risk-taking behavior. J Neurosci 35, 7226-7238.

Carskadon, M.A., Acebo, C., 1993. A self-administered rating scale for pubertal development. Journal of Adolescent Health 14, 190-195.

Cesta, C.E., Öberg, A.S., Ibrahimson, A., Yusuf, I., Larsson, H., Almqvist, C., D'Onofrio, B.M., Bulik, C.M., de la Cruz, L.F., Mataix-Cols, D., 2020. Maternal polycystic ovary syndrome and risk of neuropsychiatric disorders in offspring: prenatal androgen exposure or genetic confounding? Psychological medicine 50, 616-624.

Chapman, E., Baron-Cohen, S., Auyeung, B., Knickmeyer, R., Taylor, K., Hackett, G., 2006. Fetal testosterone and empathy: evidence from the empathy quotient (EQ) and the “reading the mind in the eyes” test. Social Neuroscience 1, 135-148.

Chen, X., Kong, L., Piltonen, T.T., Gissler, M., Lavebratt, C., 2020. Association of polycystic ovary syndrome or anovulatory infertility with offspring psychiatric and mild neurodevelopmental disorders: a Finnish population-based cohort study. Human Reproduction 35, 2336-2347.

Cruz, C.D., Pereira, O.C., 2012. Prenatal testosterone supplementation alters puberty onset, aggressive behavior, and partner preference in adult male rats. The Journal of Physiological Sciences 62, 123-131.

Dorn, L.D., 2007. Psychological and social problems in children with premature adrenarche and precocious puberty, When Puberty is Precocious. Springer, pp. 309-327.

Dorn, L.D., Susman, E.J., Ponirakis, A., 2003. Pubertal timing and adolescent adjustment and behavior: Conclusions vary by rater. Journal of youth and adolescence 32, 157-167.

Ellis, B.J., Garber, J., 2000. Psychosocial antecedents of variation in girls' pubertal timing: Maternal depression, stepfather presence, and marital and family stress. Child development 71, 485-501.

Ellis, B.J., McFadyen-Ketchum, S., Dodge, K.A., Pettit, G.S., Bates, J.E., 1999. Quality of early family relationships and individual differences in the timing of pubertal maturation in girls: a longitudinal test of an evolutionary model. Journal of personality and social psychology 77, 387.

Emberti Gialloreti, L., Mazzone, L., Benvenuto, A., Fasano, A., Garcia Alcon, A., Kraneveld, A., Moavero, R., Raz, R., Riccio, M.P., Siracusano, M., Zachor, D.A., Marini, M., Curatolo, P., 2019. Risk and Protective Environmental Factors Associated with Autism Spectrum Disorder: Evidence-Based Principles and Recommendations. Journal of Clinical Medicine 8, 217.

Engberg, H., Butwicka, A., Nordenstrom, A., Hirschberg, A.L., Falhammar, H., Lichtenstein, P., Nordenskjold, A., Frisen, L., Landen, M., 2015. Congenital adrenal hyperplasia and risk for psychiatric disorders in girls and women born between 1915 and 2010: A total population study. Psychoneuroendocrinology 60, 195-205.

Floris, D.L., Lai, M.C., Nath, T., Milham, M.P., Di Martino, A., 2018. Network-specific sex differentiation of intrinsic brain function in males with autism. Mol Autism 9, 17.

Foulkes, L., Blakemore, S.-J., 2016. Is there heightened sensitivity to social reward in adolescence? Current Opinion in Neurobiology 40, 81-85.

Gaugler, T., Klei, L., Sanders, S.J., Bodea, C.A., Goldberg, A.P., Lee, A.B., Mahajan, M., Manaa, D., Pawitan, Y., Reichert, J., 2014. Most genetic risk for autism resides with common variation. Nature genetics 46, 881-885.

Ge, X., Kim, I.J., Brody, G.H., Conger, R.D., Simons, R.L., Gibbons, F.X., Cutrona, C.E., 2003. It's about timing and change: pubertal transition effects on symptoms of major depression among African American youths. Dev Psychol 39, 430-439.

Giovanoli, S., Engler, H., Engler, A., Richetto, J., Voget, M., Willi, R., Winter, C., Riva, M.A., Mortensen, P.B., Feldon, J., 2013. Stress in puberty unmasks latent neuropathological consequences of prenatal immune activation in mice. Science 339, 1095-1099.

Goddings, A.L., Burnett Heyes, S., Bird, G., Viner, R.M., Blakemore, S.J., 2012. The relationship between puberty and social emotion processing. Developmental science 15, 801-811.

Goy, R.W., Bercovitch, F.B., McBrair, M.C., 1988. Behavioral masculinization is independent of genital masculinization in prenatally androgenized female rhesus macaques. Hormones and behavior 22, 552-571.

Greenberg, D.M., Warrier, V., Allison, C., Baron-Cohen, S., 2018. Testing the Empathizing-Systemizing theory of sex differences and the Extreme Male Brain theory of autism in half a million people. Proc Natl Acad Sci U S A 115, 12152-12157.

Grove, J., Ripke, S., Als, T.D., Mattheisen, M., Walters, R.K., Won, H., Pallesen, J., Agerbo, E., Andreassen, O.A., Anney, R., Awashti, S., Belliveau, R., Bettella, F., Buxbaum, J.D., Bybjerg-Grauholm, J., Bækvad-Hansen, M., Cerrato, F., Chambert, K., Christensen, J.H., Churchhouse, C., Dellenvall, K., Demontis, D., De Rubeis, S., Devlin, B., Djurovic, S., Dumont, A.L., Goldstein, J.I., Hansen, C.S., Hauberg, M.E., Hollegaard, M.V., Hope, S., Howrigan, D.P., Huang, H., Hultman, C.M., Klei, L., Maller, J., Martin, J., Martin, A.R., Moran, J.L., Nyegaard, M., Nærland, T., Palmer, D.S., Palotie, A., Pedersen, C.B., Pedersen, M.G., dPoterba, T., Poulsen, J.B., Pourcain, B.S., Qvist, P., Rehnström, K., Reichenberg, A., Reichert, J., Robinson, E.B., Roeder, K., Roussos, P., Saemundsen, E., Sandin, S., Satterstrom, F.K., Davey Smith, G., Stefansson, H., Steinberg, S., Stevens, C.R., Sullivan, P.F., Turley, P., Walters, G.B., Xu, X., Stefansson, K., Geschwind, D.H., Nordentoft, M., Hougaard, D.M., Werge, T., Mors, O., Mortensen, P.B., Neale, B.M., Daly, M.J., Børglum, A.D., 2019. Identification of common genetic risk variants for autism spectrum disorder. Nat Genet 51, 431-444.

Güneş, H., Tanıdır, C., Doktur, H., Önal, Z., Kutlu, E., Önal, H., Münir, K., 2020. Prenatal androgens and autistic, attention deficit hyperactivity disorder, and disruptive behavior disorders traits. Anatolian Journal of Psychiatry.

Gunnar, M.R., Wewerka, S., Frenn, K., Long, J.D., Griggs, C., 2009. Developmental changes in hypothalamus–pituitary–adrenal activity over the transition to adolescence: normative changes and associations with puberty. Development and psychopathology 21, 69.

Happé, F., Ronald, A., 2008. The 'fractionable autism triad': a review of evidence from behavioural, genetic, cognitive and neural research. Neuropsychol Rev 18, 287-304.

Herman, R., Zehr, J., Wallen, K., 2006. Prenatal androgen blockade accelerates pubertal development in male rhesus monkeys. Psychoneuroendocrinology 31, 118-130.

Herting, M.M., Uban, K.A., Gonzalez, M.R., Baker, F.C., Kan, E.C., Thompson, W.K., Granger, D.A., Albaugh, M.D., Anokhin, A.P., Bagot, K.S., Banich, M.T., Barch, D.M., Baskin-Sommers, A., Breslin, F.J., Casey, B.J., Chaarani, B., Chang, L., Clark, D.B., Cloak, C.C., Constable, R.T., Cottler, L.B., Dagher, R.K., Dapretto, M., Dick, A.S., Dosenbach, N., Dowling, G.J., Dumas, J.A., Edwards, S., Ernst, T., Fair, D.A., Feldstein-Ewing, S.W., Freedman, E.G., Fuemmeler, B.F., Garavan, H., Gee, D.G., Giedd, J.N., Glaser, P.E.A., Goldstone, A., Gray, K.M., Hawes, S.W., Heath, A.C., Heitzeg, M.M., Hewitt, J.K., Heyser, C.J., Hoffman, E.A., Huber, R.S., Huestis, M.A., Hyde, L.W., Infante, M.A., Ivanova, M.Y., Jacobus, J., Jernigan, T.L., Karcher, N.R., Laird, A.R., LeBlanc, K.H., Lisdahl, K., Luciana, M., Luna, B., Maes, H.H., Marshall, A.T., Mason, M.J., McGlade, E.C., Morris, A.S., Nagel, B.J., Neigh, G.N., Palmer, C.E., Paulus, M.P., Potter, A.S., Puttler, L.I., Rajapakse, N., Rapuano, K., Reeves, G., Renshaw, P.F., Schirda, C., Sher, K.J., Sheth, C., Shilling, P.D., Squeglia, L.M., Sutherland, M.T., Tapert, S.F., Tomko, R.L., Yurgelun-Todd, D., Wade, N.E., Weiss, S.R.B., Zucker, R.A., Sowell, E.R., 2020. Correspondence Between Perceived Pubertal Development and Hormone Levels in 9-10 Year-Olds From the Adolescent Brain Cognitive Development Study. Front Endocrinol (Lausanne) 11, 549928.

Hoekstra, R.A., Bartels, M., Cath, D.C., Boomsma, D.I., 2008. Factor structure, reliability and criterion validity of the Autism-Spectrum Quotient (AQ): a study in Dutch population and patient groups. Journal of autism and developmental disorders 38, 1555-1566.

Hollier, L.P., Mattes, E., Maybery, M.T., Keelan, J.A., Hickey, M., Whitehouse, A.J., 2013. The association between perinatal testosterone concentration and early vocabulary development: a prospective cohort study. Biological psychology 92, 212-215.

Hurst, R.M., Mitchell, J.T., Kimbrel, N.A., Kwapil, T.K., Nelson-Gray, R.O., 2007. Examination of the reliability and factor structure of the Autism Spectrum Quotient (AQ) in a non-clinical sample. Personality and Individual Differences 43, 1938-1949.

Ibáñez, L., Díaz, R., López-Bermejo, A., Marcos, M.V., 2009. Clinical spectrum of premature pubarche: links to metabolic syndrome and ovarian hyperandrogenism. Reviews in Endocrine and Metabolic Disorders 10, 63-76.

Judd, H.L., Robinson, J.D., Young, P.E., Jones, O.W., 1976. Amniotic fluid testosterone levels in midpregnancy. Obstetrics and gynecology 48, 690-692.

Kaltiala-Heino, R., Marttunen, M., Rantanen, P., Rimpelä, M., 2003. Early puberty is associated with mental health problems in middle adolescence. Social science & medicine 57, 1055-1064.

Katsigianni, M., Karageorgiou, V., Lambrinoudaki, I., Siristatidis, C., 2019. Maternal polycystic ovarian syndrome in autism spectrum disorder: a systematic review and meta-analysis. Molecular psychiatry, 1-11.

Keelan, J.A., Mattes, E., Tan, H., Dinan, A., Newnham, J.P., Whitehouse, A.J., Jacoby, P., Hickey, M., 2012. Androgen concentrations in umbilical cord blood and their association with maternal, fetal and obstetric factors. PloS one 7, e42827.

Kenyon, S., 2014. Autism in pink: Qualitative research report.

Klump, K.L., Perkins, P.S., Burt, S.A., McGue, M., Iacono, W.G., 2007. Puberty moderates genetic influences on disordered eating. Psychological Medicine 37, 627.

Knickmeyer, R., Baron-Cohen, S., Fane, B.A., Wheelwright, S., Mathews, G.A., Conway, G.S., Brook, C.G., Hines, M., 2006a. Androgens and autistic traits: A study of individuals with congenital adrenal hyperplasia. Horm Behav 50, 148-153.

Knickmeyer, R., Baron-Cohen, S., Raggatt, P., Taylor, K., 2005. Foetal testosterone, social relationships, and restricted interests in children. Journal of Child Psychology and Psychiatry 46, 198-210.

Knickmeyer, R., Baron-Cohen, S., Raggatt, P., Taylor, K., Hackett, G., 2006b. Fetal testosterone and empathy. Horm Behav 49, 282-292.

Koopman-Verhoeff, M.E., Gredvig-Ardito, C., Barker, D.H., Saletin, J.M., Carskadon, M.A., 2020. Classifying Pubertal Development Using Child and Parent Report: Comparing the Pubertal Development Scales to Tanner Staging. Journal of Adolescent Health 66, 597-602.

Kung, K.T., Spencer, D., Pasterski, V., Neufeld, S., Glover, V., O'Connor, T.G., Hindmarsh, P.C., Hughes, I.A., Acerini, C.L., Hines, M., 2016a. No relationship between prenatal androgen exposure and autistic traits: convergent evidence from studies of children with congenital adrenal hyperplasia and of amniotic testosterone concentrations in typically developing children. J Child Psychol Psychiatry 57, 1455-1462.

Kung, K.T.F., Constantinescu, M., Browne, W.V., Noorderhaven, R.M., Hines, M., 2016b. No relationship between early postnatal testosterone concentrations and autistic traits in 18 to 30-month-old children. Molecular Autism 7, 15.

Kung, K.T.F., Thankamony, A., Ong, K.K.L., Acerini, C.L., Dunger, D.B., Hughes, I.A., Hines, M., 2021. No relationship between prenatal or early postnatal androgen exposure and autistic traits: evidence using anogenital distance and penile length measurements at birth and 3 months of age. J Child Psychol Psychiatry 62, 876-883.

Lai, M.C., Lombardo, M.V., Suckling, J., Ruigrok, A.N., Chakrabarti, B., Ecker, C., Deoni, S.C., Craig, M.C., Murphy, D.G., Bullmore, E.T., Baron-Cohen, S., 2013. Biological sex affects the neurobiology of autism. Brain 136, 2799-2815.

Lakens, D., 2017. Equivalence Tests: A Practical Primer for t Tests, Correlations, and Meta-Analyses. Soc Psychol Personal Sci 8, 355-362.

Lee, J.K., Amaral, D.G., Solomon, M., Rogers, S.J., Ozonoff, S., Nordahl, C.W., 2020. Sex Differences in the Amygdala Resting-State Connectome of Children With Autism Spectrum Disorder. Biological Psychiatry: Cognitive Neuroscience and Neuroimaging 5, 320-329.

Lombardo, M.V., Auyeung, B., Pramparo, T., Quartier, A., Courraud, J., Holt, R.J., Waldman, J., Ruigrok, A.N., Mooney, N., Bethlehem, R.A., 2018. Sex-specific impact of prenatal androgens on social brain default mode subsystems. Molecular psychiatry, 1-14.

Loomes, R., Hull, L., Mandy, W.P.L., 2017. What is the male-to-female ratio in autism spectrum disorder? A systematic review and meta-analysis. Journal of the American Academy of Child & Adolescent Psychiatry 56, 466-474.

Lutchmaya, S., Baron-Cohen, S., Raggatt, P., 2001. Foetal testosterone and vocabulary size in 18-and 24-month-old infants. Infant Behavior and Development 24, 418-424.

Lutchmaya, S., Baron-Cohen, S., Raggatt, P., 2002. Foetal testosterone and eye contact in 12-month-old human infants. Infant Behavior and Development 25, 327-335.

Marceau, K., Kirisci, L., Tarter, R.E., 2019. Correspondence of pubertal neuroendocrine and Tanner stage changes in boys and associations with substance use. Child development 90, e763-e782.

May, T., Yi, K.L.J., Loveland, K.L., Vollenhoven, B., Williams, K., 2021. Overlap of autism and conditions associated with atypical sex hormone levels or response: A systematic review and meta-analysis. Research in Autism Spectrum Disorders 80, 101693.

McGovern, C.W., Sigman, M., 2005. Continuity and change from early childhood to adolescence in autism. J Child Psychol Psychiatry 46, 401-408.

Mendle, J., Harden, K.P., Brooks-Gunn, J., Graber, J.A., 2010. Development's tortoise and hare: pubertal timing, pubertal tempo, and depressive symptoms in boys and girls. Developmental Psychology 46, 1341.

Ng, V.K., Cribbie, R.A., 2017. Using the gamma generalized linear model for modeling continuous, skewed and heteroscedastic outcomes in psychology. Current Psychology 36, 225-235.

Padmanabhan, V., Manikkam, M., Recabarren, S., Foster, D., 2006. Prenatal testosterone excess programs reproductive and metabolic dysfunction in the female. Molecular and cellular endocrinology 246, 165-174.

Park, B.Y., Lee, B.K., Burstyn, I., Tabb, L.P., Keelan, J.A., Whitehouse, A.J., Croen, L.A., Fallin, M.D., Hertz-Picciotto, I., Montgomery, O., 2017. Umbilical cord blood androgen levels and ASD-related phenotypes at 12 and 36 months in an enriched risk cohort study. Molecular autism 8, 3.

Peper, J.S., Brouwer, R.M., van Baal, G.C.M., Schnack, H.G., van Leeuwen, M., Boomsma, D.I., Kahn, R.S., Pol, H.E.H., 2009. Does having a twin brother make for a bigger brain? European Journal of Endocrinology 160, 739-746.

Petersen, A.C., Crockett, L., Richards, M., Boxer, A., 1988. A self-report measure of pubertal status: Reliability, validity, and initial norms. Journal of youth and adolescence 17, 117-133.

Picci, G., Scherf, K.S., 2015. A two-hit model of autism: Adolescence as the second hit. Clinical Psychological Science 3, 349-371.

Risal, S., Pei, Y., Lu, H., Manti, M., Fornes, R., Pui, H.-P., Zhao, Z., Massart, J., Ohlsson, C., Lindgren, E., 2019. Prenatal androgen exposure and transgenerational susceptibility to polycystic ovary syndrome. Nature medicine 25, 1894-1904.

Rodeck, C., Gill, D., Rosenberg, D., Collins, W., 1985. Testosterone levels in midtrimester maternal and fetal plasma and amniotic fluid. Prenatal diagnosis 5, 175-181.

Romeo, R.D., 2003. Puberty: a period of both organizational and activational effects of steroid hormones on neurobehavioural development. Journal of neuroendocrinology 15, 1185-1192.

Roselli, C.E., Estill, C.T., Stadelman, H.L., Meaker, M., Stormshak, F., 2011. Separate Critical Periods Exist for Testosterone-Induced Differentiation of the Brain and Genitals in Sheep. Endocrinology 152, 2409-2415.

Sawyer, S.M., Azzopardi, P.S., Wickremarathne, D., Patton, G.C., 2018. The age of adolescence. The Lancet Child & Adolescent Health 2, 223-228.

Schielzeth, H., Dingemanse, N.J., Nakagawa, S., Westneat, D.F., Allegue, H., Teplitsky, C., Réale, D., Dochtermann, N.A., Garamszegi, L.Z., Araya‐Ajoy, Y.G., Sutherland, C., 2020. Robustness of linear mixed‐effects models to violations of distributional assumptions. Methods in Ecology and Evolution 11, 1141-1152.

Schindler, A.E., 2012. Hormones in human amniotic fluid.

Schmitz, K.E., Hovell, M.F., Nichols, J.F., Irvin, V.L., Keating, K., Simon, G.M., Gehrman, C., Jones, K.L., 2004. A Validation Study of Early Adolescents’ Pubertal Self-Assessments. The Journal of Early Adolescence 24, 357-384.

Schulz, K.M., Molenda-Figueira, H.A., Sisk, C.L., 2009. Back to the future: The organizational-activational hypothesis adapted to puberty and adolescence. Horm Behav 55, 597-604.

Shirtcliff, E.A., Dahl, R.E., Pollak, S.D., 2009. Pubertal development: correspondence between hormonal and physical development. Child development 80, 327-337.

Siegel, J.M., Yancey, A.K., Aneshensel, C.S., Schuler, R., 1999. Body image, perceived pubertal timing, and adolescent mental health. Journal of adolescent health 25, 155-165.

Smith, J.T., Waddell, B.J., 2000. Increased fetal glucocorticoid exposure delays puberty onset in postnatal life. Endocrinology 141, 2422-2428.

Smith, R.E., Avery, J.A., Wallace, G.L., Kenworthy, L., Gotts, S.J., Martin, A., 2019. Sex differences in resting-state functional connectivity of the cerebellum in autism spectrum disorder. Frontiers in human neuroscience 13, 104.

Smith-Woolley, E., Rimfeld, K., Plomin, R., 2017. Weak associations between pubertal development and psychiatric and behavioral problems. Transl Psychiatry 7, e1098-e1098.

Stratis, E.A., Lecavalier, L., 2015. Informant agreement for youth with autism spectrum disorder or intellectual disability: a meta-analysis. J Autism Dev Disord 45, 1026-1041.

Tan, D.W., Gilani, S.Z., Maybery, M.T., Mian, A., Hunt, A., Walters, M., Whitehouse, A.J.O., 2017. Hypermasculinised facial morphology in boys and girls with Autism Spectrum Disorder and its association with symptomatology. Sci Rep 7, 9348.

Tan, D.W., Maybery, M.T., Ewing, L., Tay, J.X., Eastwood, P.R., Whitehouse, A.J.O., 2020a. Sex-specific variation in facial masculinity/femininity associated with autistic traits in the general population. Br J Psychol 111, 723-741.

Tan, D.W., Maybery, M.T., Gilani, S.Z., Alvares, G.A., Mian, A., Suter, D., Whitehouse, A.J.O., 2020b. A broad autism phenotype expressed in facial morphology. Transl Psychiatry 10, 7.

Tsompanidis, A., Aydin, E., Padaigaitė, E., Richards, G., Allison, C., Hackett, G., Austin, T., Holt, R., Baron-Cohen, S., 2021. Maternal steroid levels and the autistic traits of the mother and infant. Molecular Autism 12, 51.

van de Beek, C., Thijssen, J.H., Cohen-Kettenis, P.T., van Goozen, S.H., Buitelaar, J.K., 2004. Relationships between sex hormones assessed in amniotic fluid, and maternal and umbilical cord serum: what is the best source of information to investigate the effects of fetal hormonal exposure? Hormones and Behavior 46, 663-669.

Warrier, V., Toro, R., Won, H., Leblond, C.S., Cliquet, F., Delorme, R., De Witte, W., Bralten, J., Chakrabarti, B., Børglum, A.D., 2019. Social and non-social autism symptoms and trait domains are genetically dissociable. Communications biology 2, 1-13.

Whitehouse, A.J., Mattes, E., Maybery, M.T., Dissanayake, C., Sawyer, M., Jones, R.M., Pennell, C.E., Keelan, J.A., Hickey, M., 2012a. Perinatal testosterone exposure and autistic-like traits in the general population: a longitudinal pregnancy-cohort study. Journal of Neurodevelopmental Disorders 4, 25.

Whitehouse, A.J., Mattes, E., Maybery, M.T., Sawyer, M.G., Jacoby, P., Keelan, J.A., Hickey, M., 2012b. Sex‐specific associations between umbilical cord blood testosterone levels and language delay in early childhood. Journal of Child Psychology and Psychiatry 53, 726-734.

Wichstrøm, L., 2000. Predictors of adolescent suicide attempts: a nationally representative longitudinal study of Norwegian adolescents. Journal of the American Academy of Child & Adolescent Psychiatry 39, 603-610.

Xiong, H., Scott, S., 2020. Amniotic testosterone and psychological sex differences: A systematic review of the extreme male brain theory. Developmental Review 57, 100922.
